# Design of High-Surface-Area Bimetallic Ag–Cu Nanostructures with a Tunable Ratio Obtained via Selective Leaching of AlAgCu Alloys

**DOI:** 10.1021/acs.jpcc.5c02677

**Published:** 2025-07-24

**Authors:** Maaike E. T. Vink - van Ittersum, Masoud Lazemi, Remco Dalebout, Johannes D. Meeldijk, Matt L. J. Peerlings, Juliette C. Verschoor, Bianca Ligt, Emiel Hensen, Ad van der Eerden, Peter Ngene, Petra E. de Jongh

**Affiliations:** † Materials Chemistry & Catalysis, Debye Institute for Nanomaterials Science, 8125 Utrecht University, Universiteitsweg 99, Utrecht 3584CG, The Netherlands; ‡ Inorganic Materials & Catalysis, Chemical Engineering and Chemistry, Eindhoven University of Technology, Eindhoven 5600 MB, The Netherlands

## Abstract

Nanostructured metals are promising for applications as energy materials. Often, several metal components must be combined to obtain the desired properties. However, preparing high-surface-area bimetallic metals with a desired spatial distribution can be challenging. We developed a novel synthesis route to make nanostructured Ag_*x*_Cu_10‑x_ with control over the Ag:Cu molar ratio, covering the full range from *x* = 0 to *x* = 10. We used a dealloying synthesis route based on leaching Al from an AlAgCu mixed phase. We introduced a quenching step after alloying and before leaching to suppress the formation of side phases, which is beneficial for the leaching step. High-surface-area AgCu samples with a tunable Ag:Cu ratio and Ag and Cu mixed on tens of nanometer scale were obtained. The AgCu samples were applied as catalysts in the electrochemical reduction of CO_2_, showing a clear dependence of the selectivity on the Cu content. An optimum in C_2_H_4_ production was found for a Cu content between 50 and 70 atom % in the nanostructures. After catalysis, the molar ratios had not changed significantly, showing the stability of these catalysts. This work shows the usefulness of a method to prepare nanostructured catalyst covering the full Ag:Cu ratio.

## Graphical abstract

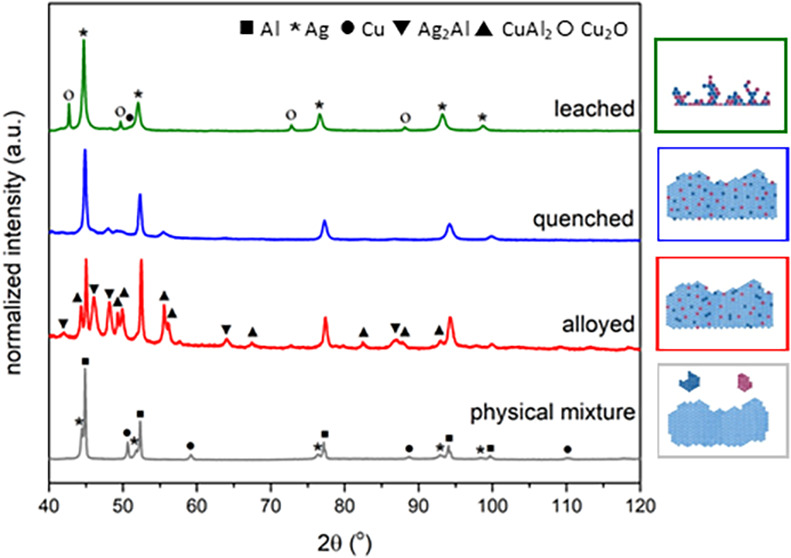

## 1 Introduction

As heterogeneous catalytic reactions take place at the surface of a catalyst, it is beneficial for the activity to have a catalyst with a high surface area.^1^ This can, for example, be achieved not only by placing nanoparticles on a support but also by the use of porous metals. A famous example of the latter is the Raney nickel catalyst that is used for hydrogenation reactions.^2^ This catalyst is prepared via selective Al leaching from a NiAl alloy. This dealloying synthesis route has been used to prepare a broad range of porous metals, among other porous Au, Ag, and Cu.^3–10^ These materials are very useful in (electro)­catalysis and energy storage applications due to the absence of ligands and their bulk nature, which leads to good electronic conductivity. Also, the high control over the synthesis and the resulting structuring makes them highly interesting.^11^

To improve the selectivity of a catalyst, bimetallic catalysts are highly interesting. The use of bimetallics can either change the intrinsic activity via electronic or geometric effects, or lead to tandem catalysis.^12–14^ In tandem catalysis, different steps of the reaction take place at different sites in the catalyst. The right intimacy of the two active sites is highly important for the catalysts to steer the product selectivity in the right direction.^12,14,15^

Using the dealloying synthesis route, bimetallic porous metals like AgCu and AuCu have been made.^16–25^ For porous AgCu, it is known that it can be made starting from several alloys, *e.g*., AgCuZr, AgCuSn, AgCuZn, and AgCuAl.^16,18–22,24^ Most often, these structures have Ag and Cu at scale intimacies of a few nanometer down to atomic intimacy.^16,18,20–22,24,25^ Sometimes, larger (Cu) particles are present too.^23,25^ Ag and Cu at nanoscale intimacies have not been mentioned. To our knowledge, no systematic variation of the Ag:Cu ratio has been reported either.

In this work, we designed a synthesis route based on the dealloying principle. Starting from a trimetallic AlAgCu alloy, we were able to synthesize AgCu mixtures with a high surface area and tens of nanometer Ag and Cu intimacy. The AgCu mixtures with varying Ag:Cu ratios over the full molar ratio range were applied as catalysts in a model reaction, the electrochemical reduction of CO_2_.

## 2 Experimental Section

### 2.1 Synthesis

First, five metal mixtures were made with Al:Ag:Cu molar ratios of 90:10:0, 90:7:3, 90:5:5, 90:3:7, and 90:0:10. These were made by thoroughly grinding Ag (99.9%, APS 1.3–3.2 μm, Alfa Aesar), Cu (99.9%, APS 3.25–4.75, Alfa Aesar), and Al (99.5%, APS 7–15 μm, Alfa Aesar) with a mortar and pestle in an Ar-filled glovebox (O_2_ < 0.1 ppm, H_2_O < 1 ppm). The molar ratios of the samples can be found in Table 1. The aim was to prepare ∼0.8 g of high-surface-area AgCu. The mixtures (∼2.5–4 g) were heated to 1100 °C with a ramp of 10 °C/min in a tubular oven (Thermo Scientific F79340–33) under an argon flow (600 mL/min) to alloy the metals. The oven was kept at 1100 °C for 2 h and subsequently allowed to cool down to room temperature.

**Table 1:** Composition of the 5 Al_90_Ag_*x*_Cu_10‑x_ Starting Alloys

sample	mole Al	mole Ag	mole Cu
Al_90_Ag_10_	90	10	0
Al_90_Ag_7_Cu_3_	90	7	3
Al_90_Ag_5_Cu_5_	90	5	5
Al_90_Ag_3_Cu_7_	90	3	7
Al_90_Cu_10_	90	0	10

When the samples were fully cooled, a quenching step was performed. The samples were heated to 560 °C with a ramp of 10 °C/min in a Nabertherm muffle oven and kept at this temperature for 4 h. After this, the samples were directly quenched with cold tap water. Then, the metal mixtures were left to dry in the air.

To obtain the high-surface-area AgCu structures with varying AgCu ratios, Al was leached by placing the sample in 350 mL of a 1 M HCl solution (37%, EMSURE). After 45 h, the bubble formation (due to the formation of H_2_ from the reaction between HCl and Al) had stopped for all five samples and the metal mixtures were washed with Milli-Q until the pH of the solution became neutral. Then, the mixtures were dried in an oven in static air at 80 °C for 2.5 h.

To make electrodes for catalysis, an ink was made by mixing 10 mg of catalyst powder, 4470 μL of Milli-Q, 1120 μL of isopropanol (99.5%, Sigma-Aldrich), and 44 μL of Nafion D-520 dispersion (5 wt %, ≥1.00 mequiv/g exchange capacity, Sigma-Aldrich) and subsequent sonication for 1 h. Then, it was drop cast on a sheet of carbon paper (Toray TGP-H-060) and heated on a heating plate at 80 °C to accelerate the drying process.

### 2.2 Characterization

X-ray diffraction (XRD) was measured on a Bruker D2 Phaser with a Co Kα X-ray source (1.79028 Å) and on a Bruker D2 Phaser with a Cu Kα X-ray source (1.54187 Å). The angles of the diffractogram (*x*-axis) from the latter were converted to angles from a Co Kα source using Bragg’s law. Diffractograms were measured between 40 and 120° with a step size of 0.03° and a dwell time of 1 s per step. For the porous Ag_*x*_Cu_1–*x*_ sample, a second diffractogram was measured between 86 and 94° with a step size of 0.01° and a dwell time of 1 s per step. All diffraction data was normalized. As the metal mixtures after alloying and after quenching consisted of a single large piece, the sample thicknesses were reduced to fit in the XRD holder by pressing with a press (max. Four ktons of pressure) and rolling with a rolling mill the sample to a thickness of 2 mm or smaller before measuring the X-ray diffractogram.

The Bruker DIFFRAC.SUITE TOPAS software was used to perform Rietveld refinement. The crystallographic data of the Al (cubic), Ag (cubic), Cu (cubic), Ag_2_Al (hexagonal), CuAl_2_ (tetragonal), Cu_2_O (cubic), and CuO (monoclinic) phases between 40 and 120° 2θ (Co source) or between 33 and 97° 2θ (Cu source) was fitted using the Lorentzian function for its peaks. No strain was incorporated in the lattice planes, but preferred orientations were used as expected for the nonpowdered samples. The background was fitted with a Chebyshev polynomial with *n* ≤ 3. The composition based on the crystalline phases present was obtained after the fitting reached a minimum goodness-of-fit (GOF) value.

Scanning electron microscopy (SEM) images were taken on a Thermo Fisher Scientific Helios G3 UC, operated at 10 or 15 kV and 50 pA. Energy-dispersive X-ray (EDX) spectroscopy measurements were performed using an Oxford Instruments X-Max^N^ 150 mm^2^ detector. Scanning transmission electron microscopy (STEM) in combination with EDX was performed on a Talos F200X at 200 kV with a Super XG1 EDX detector in a low-background TEM holder using the net signal in the Velox software. TEM samples were prepared by dispersing the catalyst powder in ethanol. After a few minutes, a few droplets of the partially precipitated supernatant were dropped on a Ni grid with a homemade holey polymer film.

Nitrogen physisorption experiments were performed on a Micromeritics TriStar II Plus after drying the samples under a vacuum at 80 °C overnight. Measurements were performed using liquid N_2_ at 77 K. The BET surface area was determined by fitting the isotherms using the Rouquerol criteria.^26^ The total pore volume was the adsorbed volume at a relative pressure of *p*/*p*_0_ ≈ 0.995. The BJH analysis was applied to the adsorption branch of the physisorption data to extract the pore size distributions,^27^ and the t-plot was used to obtain the microporosity.

Inductively coupled plasma (ICP) was measured by a Mikroanalytisches Labor Kolbe (Mikroanalytisches Labor Kolbe c/o Fraunhofer Institut UMSICHT, Germany) to determine the amounts of Ag, Cu, Al, and C. The values for C were determined using an Elementar Model Vario Mikro CHNS analyzer. The elements Ag, Cu, and Al were measured on a Spectro Model Spectro Arcos ICP after microwave digestion on a CEM MARS 6.

X-ray photoelectron spectroscopy (XPS) was measured at the Eindhoven University of Technology on a K-α ultrahigh vacuum X-ray photoelectron spectrometer (Thermo Fisher Scientific) using a monochromatic aluminum anode (Kα = 1486.6 eV, 72 W) X-ray source with a spot size of 400 μm. The samples were measured with a pass energy of 50 eV. CasaXPS software was used to analyze and fit the data. For the quantitative analysis, cross sections were considered. The Ag 3d peaks and Cu 2p peaks were used together with the Ag MNN and Cu LMM peaks to determine which phases were present.

### 2.3 Electrochemical Performance

The electrochemical measurements were performed in a custom-made H-cell,^28^ which consisted of a commercial iridium oxide-based anode (Dioxide Materials), a 3 M Ag/AgCl reference electrode (Metrohm), and our catalyst on top of a glassy carbon disc (SIGRADUR K) as the cathode. The anodic and cathodic compartments (both 18 mL) were separated by a Fumasep anion exchange membrane (Fumasep FAA-3-PK-130, Fumatech BWT GmbH) and were filled with 15 mL of 0.1 M KHCO_3_ electrolyte (Honeywell Fluka, 99.7%). On the cathodic side, a CO_2_ flow of 10 mL/min was applied, while on the anodic side, an Ar flow of 10 mL/min was used. Both sides were stirred with a stirring bar at 500 rpm.

Before any measurement, the catholyte was saturated with CO_2_ for at least 0.5 h. Electrochemical impedance spectroscopy (EIS) was measured to determine the uncompensated resistance. The Nyquist plot was fitted with an R1+Q2/R2 equiv circuit, which gave the uncompensated resistance (R1). The resistance was used for an *iR* correction of 80% during the measurement, and the remainder was corrected during data analysis.

A cyclic voltammogram (*C–V*) was recorded between 0.3 V and −1.0 V vs RHE with a scan rate of 0.01 V/s for 3 cycles to reduce any oxide present. Then, the double-layer capacitance was determined by measuring CVs between 0.3 V and −0.2 V vs RHE for 4 cycles with scan rates between 0.02 and 1.4 V/s. The averaged current from the last 3 cycles at 0.05 V vs RHE was plotted versus the scan rate. The slope of a linear fit in the linear regime of this plot was used to determine the capacitance of the electrode.

Lastly, catalysis was measured by chronoamperometry (CA) at subsequently −0.4, −0.6, −0.8, and −1.0 V vs RHE. After each potential, a liquid sample of 1 mL was taken and replenished with 1 mL of fresh electrolyte. The presence of formate and ethanol was quantified with ^1^H NMR, using a 400 MHz Varian NMR with solvent suppression and 50 mM phenol and 10 mM DMSO as an internal standard solution. Gaseous products were online detected by a Gas Analyzer Solution Compact Microcompact GC 4.0 gas chromatograph. This GC had three columns: a Rt-QBond (10 m · 0.32 mm, Agilent), a Molecular Sieve 5A (10 m · 0.53 mm, Restek), and a Carboxen 1010 (8 m · 0.32 mm, Agilent) column, connected with, respectively, an FID detector, an FID detector (together with methanizer to increase the CO sensitivity), and a TCD detector to measure the presence of CH_4_, C_2_H_4_, and C_2_H_6_ (first column), CO and CH_4_ (second column), and H_2_ and CO_2_ (third column).

## 3 Results and Discussion

### 3.1 From Single-Phase Trimetallic AlAgCu to Bimetallic AgCu

To develop a synthesis route to make high-surface-area bimetallic AgCu nanostructures with tunable Ag:Cu ratios, a dealloying strategy was explored. The investigated synthesis route is schematically depicted in Figure 1. First, pure metals (Figure 1, step 1) were mixed and subsequently heat-treated at 1100 °C (and cooled to room temperature) to make an alloy (Figure 1, step 2). In Figure 2, the diffractograms of a physical mixture (gray) and an alloyed mixture (red) are given for the Al_90_Ag_5_Cu_5_ sample with an Al:Ag:Cu atomic ratio of 90:5:5. For the physical mixture, not only the most dominant peaks are from Al (45, 53, 77, 94, and 100°) but also peaks from Ag (45, 52, 77, 93 and 99°) and Cu (51, 59, 89 and 110°) are present. Rietveld refinement (Table S1 and Figure S1) was used to determine the composition of the crystalline phases, which consisted of 84.6 atom % Al, 9.2 atom % Ag, and 6.2 atom % Cu. This is a slightly higher Ag and Cu content than intended.

**Figure 1:**
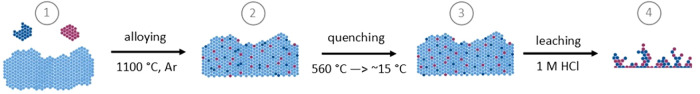
Schematic overview of the synthesis route of porous Ag_*x*_Cu_10‑x_ nanostructures starting from a physical mixture of Ag, Cu, and Al (1), which are alloyed at 1100 °C (2), quenched from 560 to ∼15 °C (3), and leaching the Al in a 1 M HCl solution (4).

**Figure 2:**
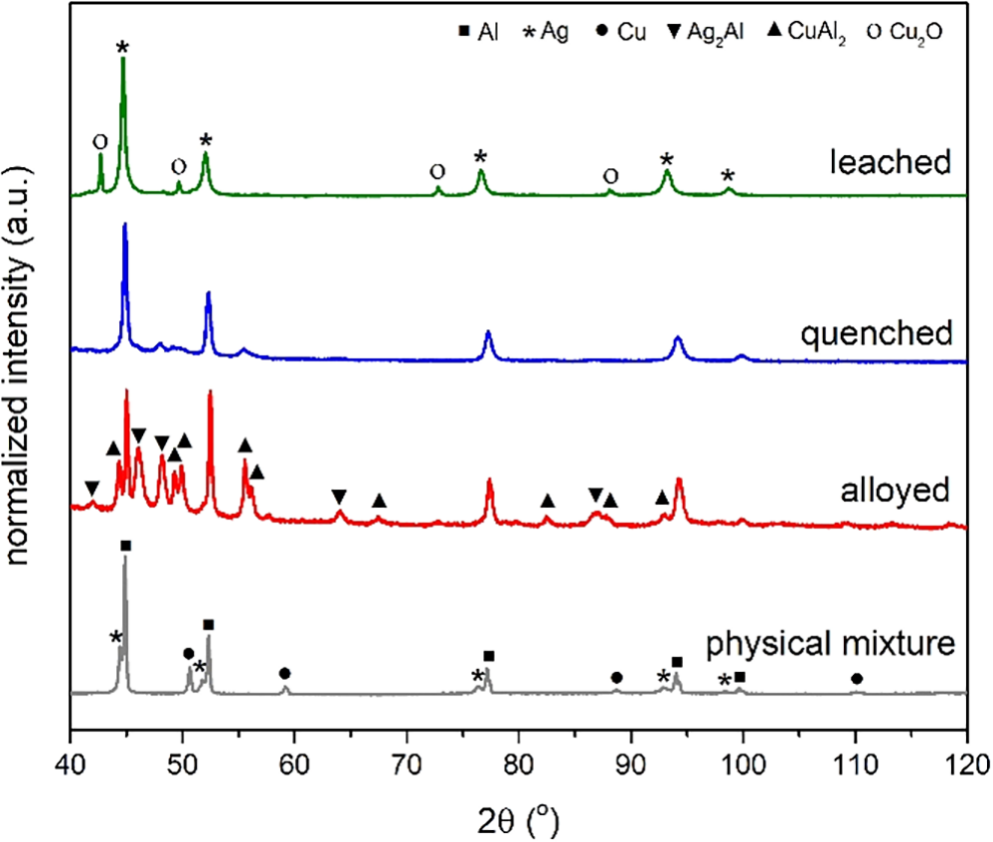
X-ray diffractogram of the physical mixture of Al_90_Ag_5_Cu_5_, the alloyed Al_90_Ag_5_Cu_5_ mixture, the quenched Al_90_Ag_5_Cu_5_ mixture, and porous Ag_5_Cu_5_ after leaching.

After the heat treatment at 1100 °C, the peaks of Ag and Cu have disappeared (red line), while the Al peaks are still present. Based on the phase diagrams of Al–Ag–Cu, Al–Cu, and Al–Ag, this is likely not a pure Al phase but an Al phase with both Ag and Cu dissolved in it.^29–32^ This can be verified by looking at the exact position of the Al peak, as metallic Al, Ag, and Cu have an FCC lattice structure. For example, for pure Al, the (311) peak is found at 94.2°. The presence of Ag and Cu changed this peak position. The presence of Ag, which has its (311) peak at 93.2°, leads to a peak at smaller 2Θ values. By contrast, the presence of Cu, which has its (311) peak at 110.3°, leads to larger 2Θ values. The theoretical peak position is just a weighted average of the individual peak positions, assuming all Ag and Cu atoms are in this phase and, hence, no other phases are formed. In that case, the peak should be at 95.0° for the Al_90_Ag_5_Cu_5_ sample. The actual peak is found at 94.3°, implying that there is slightly less Cu in this phase present. This can be explained by the presence of the CuAl_2_ and Ag_2_Al phases of, respectively, 14.8 and 3.4 atom % (Table S1 and Figure S1).^3,29,30^ Since the amount of Cu in the CuAl_2_ phase is higher than the amount of Ag in the Ag_2_Al phase, this explains the shift in the Al (311) peak.

Dealloying works best when one single alloyed phase is present.^3^ To suppress the formation of CuAl_2_ and Ag_2_Al phases, we added a quenching step (Figure 1, step 3). From the literature, it is known that for AlAg alloys, the Ag_2_Al phase can be suppressed by heating the alloy at 560 °C and subsequent quenching.^3,5^ This traps Al and Ag in a metastable phase, preventing the formation of the Ag_2_Al phase. This procedure was explored for the Al_90_Ag_5_Cu_5_ alloy. The blue line in Figure 2 shows the diffractogram after quenching. Clearly, the peaks related to the Ag_2_Al and CuAl_2_ phases are suppressed after quenching. The Ag_2_Al phase is decreased from 3.4 to 0.9 atom %, and the CuAl_2_ phase is decreased from 14.8 to 4.4 atom % of the crystalline phases. Also, the peaks of Al have broadened, implying that crystallites with slightly larger and smaller *d* values are present. This indicates that Ag and Cu are almost fully mixed into the Al phase. Therefore, we have proven that also for AlAgCu alloys, quenching is a useful step to suppress undesired phases.

Having a single AlAgCu phase, we moved on to the dealloying step, where the Al was leached out of the structure by using HCl (Figure 1, step 4). For the Al_90_Ag_5_Cu_5_ alloy, this led to a porous sample named p-Ag_5_Cu_5_. The green line in Figure 2 shows the diffractogram after leaching out Al with peaks of Ag (45, 52, 77, 93, and 99°) and Cu_2_O (43, 50, 73, and 88°). Rietveld refinement showed that 96.6% of the crystalline phases is Ag and only 3.4% is Cu_2_O. The Ag content is higher than was intended. However, it cannot be excluded that noncrystalline phases are present, causing these differences. The most intense Ag peak (45°) was found at slightly larger 2Θ values than in the physical mixture, which is ascribed to some residual Al in the Ag phase. We discuss the exact composition in more detail below. The diffractogram does not show any peaks related to Al, Ag_2_Al, or CuAl_2_, indicating that no detectable Al crystallites remain present after leaching. Therefore, we propose the synthesis route of subsequent alloying, quenching, and dealloying, which is schematically depicted in Figure 1 as the synthesis route for dealloyed AgCu.

### 3.2 A Tunable Ag:Cu Composition

To investigate if this synthesis route can yield nanostructures with a broad range of Ag:Cu ratios, dealloyed AgCu samples with five varying Ag:Cu ratios were made by using the synthesis described above. The atomic ratios are given in Table 1, and the porous samples were named after their molar ratio: p-Ag, p-Ag_7_Cu_3_, p-Ag_5_Cu_5_, p-Ag_3_Cu_7_, and p-Cu. For all samples, similar trends in the XRD patterns (Figure S2) were found as previously described for the p-Ag_5_Cu_5_ sample.

It is interesting to compare the XRD patterns of these leached samples, which are given in Figure 3A. The diffractograms of p-Ag (blue) and p-Cu (orange) show peaks related to Ag (45, 52, 77, 93, and 99°), Cu (51°), Cu_2_O (43, 50, 73, and 88°), and CuO (42, 45, 57, 63, 69, 78, 79, 81, and 90°). The diffractograms of p-Ag_7_Cu_3_ (violet), p-Ag_5_Cu_5_ (purple), and p-Ag_3_Cu_7_ (brown) show mixtures of Ag, Cu, and Cu_2_O peaks. To understand the composition of the samples better, Figure 3B zooms in on the diffractograms between 86 and 94°. In this region lies the Ag (311) peak at 93.1° and the Cu (220) peak at 88.8° and/or the Cu_2_O (311) peak at 88.1°. For all bimetallic AgCu samples, the Ag (311) peak is slightly shifted to higher 2Θ values, which is an indication that there is either some Al or some Cu in this phase. Similarly, the Cu_2_O peaks are slightly shifted to higher 2Θ values. These results could be explained by the presence of a metastable alloyed phase of Ag containing Cu and *vice versa*.^33^ This will be discussed in more detail below. The ratio of the Ag-based and Cu-based phases (Table 2; based on the results in Table S1) scales with the amount of Ag and Cu present. However, the Ag content is much higher than intended, which could be caused by the presence of noncrystalline phases that are rich in Cu.

**Figure 3:**
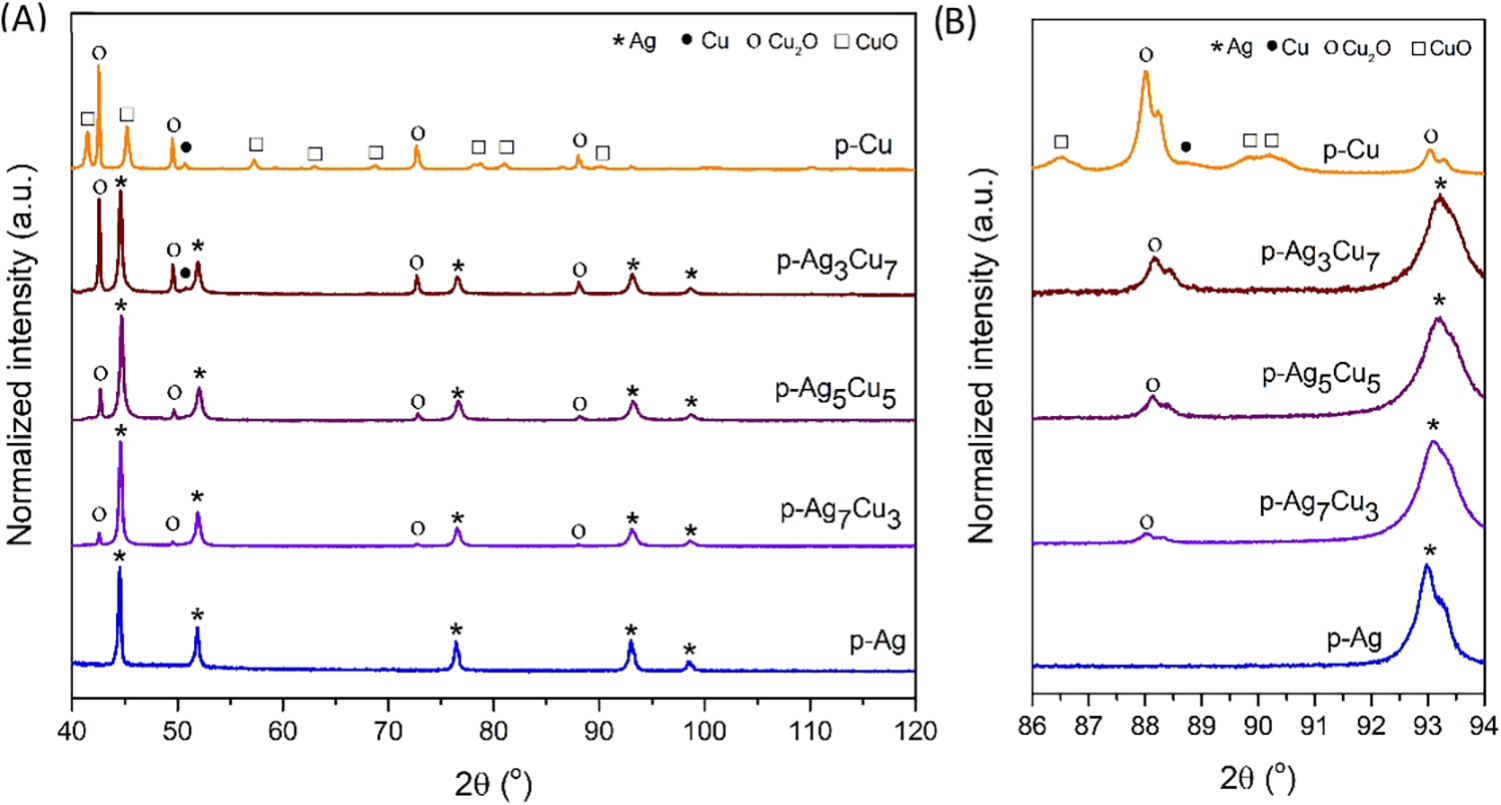
X-ray diffractogram of p-Ag, p-Ag_7_Cu_3_, p-Ag_5_Cu_5_, p-Ag_3_Cu_7_, and p-Cu for 2Θ values (A) from 40 to 120°; and (B) a zoom-in from 86 to 94°.

**Table 2:** Ag:Cu Molar Ratio Based on XRD, ICP, and XPS Data, and Pore Volume and Surface Area Derived from N_2_ Physisorption Results for the Five Porous Ag_*x*_Cu_10‑x_ Samples

sample	Ag:Cu ratio (theoretical)	Ag:Cu ratio (XRD)	Ag:Cu ratio (ICP)	Ag:Cu ratio (XPS)	pore volume (cm^3^/g)	BET surface area (m^2^/g)
p-Ag					0.01	3
p-Ag_7_Cu_3_	2.33	62.00	2.42	0.97	0.04	9
p-Ag_5_Cu_5_	1.00	14.21	1.10	0.69	0.11	25
p-Ag_3_Cu_7_	0.43	1.77	0.43	0.31	0.08	23
p-Cu					0.03	12

As the XRD peak positions and intensities suggest tunability of the Ag:Cu ratio, but a large deviation in crystalline Cu content was found, inductively coupled plasma (ICP) was employed to investigate also the presence of noncrystalline Cu. The raw data can be found in Table S2, and the Ag:Cu ratios are given in Table 2 as well as the theoretical Ag:Cu ratios. The raw data showed that some Al is still present in the samples. From the literature, it is known that alloys never completely dealloy due to limited electrolyte penetration and surface restructuring that is faster than metal dissolution.^11,34,35^ For the p-Ag_3_Cu_7_ sample, the Ag:Cu ratio exactly matched the theoretical ratio. For the p-Ag_7_Cu_3_ and p-Ag_5_Cu_5_ samples, slightly more Ag was found than predicted. Possibly, a small part of the Cu has leached out too. However, the differences were small (<10% deviation).

As both XRD and ICP provide information about the bulk material, X-ray photoelectron spectroscopy (XPS) was used to see if there are any differences between the bulk and surface compositions of the dealloyed samples. In Figure S3, the XPS spectra for the five p-AgxCu10_–*x*_ samples are given. Figure 4A,B zooms in on, respectively, the Ag 3d and Cu 2p peaks. The Ag 3d peaks (around 368 and 374 eV) become smaller, and the Cu 2p peaks (around 932 eV) become more prominent when going from the p-Ag to the p-Cu samples. Fitting the Ag 3d and Cu 2p peaks (Figure S4) based on the peak positions found in the literature together with the Ag MNN peak and Cu LMM peaks (Figure S4) to determine the oxide phases present^36–42^ gave the atomic Ag:Cu ratios given in Table 2. As the ratios are lower than the ratios found with ICP, especially for the Ag-rich samples, this suggests that there is slightly less Cu present in the bulk than on the surface. This is likely explained by the synthesis route, where the less noble atoms (Cu) are leached starting from the surface and then moving inside. As Cu is less noble than Ag, it will move faster from the bulk to the surface (and maybe even partially leach out). In theory, it cannot be excluded that surface segregation plays a role, as well. However, based on the literature, we would expect a higher Ag content on the surface for AgCu mixtures.^43^ Still, the ratios from ICP nicely match the intended ratios. Hence, we conclude that this selective leaching synthesis route is very suitable for the synthesis of nanostructured AgCu with a well-defined Ag:Cu ratio.

**Figure 4:**
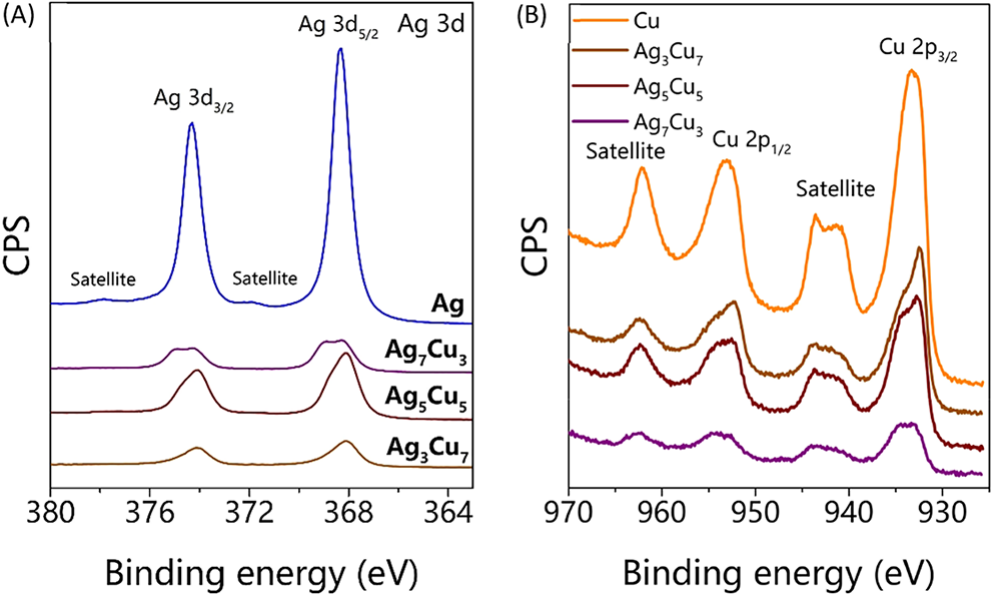
X-ray photoelectron spectroscopy of p-Ag, p-Ag_7_Cu_3_, p-Ag_5_Cu_5_, p-Ag_3_Cu_7_, and p-Cu showing (A) the Ag 3d_3/2_ and Ag 3d_5/2_ peaks and (B) the Cu 2p_1/2_ and Cu 2p_3/2_ peaks.

### 3.3 High-Surface-Area AgCu with Nanoscale Intimacy

Although the XRD and ICP data provide much information about the composition of the samples, they do not give any information about the morphology of the sample. Figure 5 gives the scanning electron microscopy (SEM) image of the p-Ag_5_Cu_5_ sample. Clearly, this sample is nanostructured. Similar nanostructures were found for the p-Ag, p-Ag_7_Cu_3_, p-Ag_3_Cu_7_, and p-Cu samples (Figure S5). This shows that it is possible to make porous bimetallic AgCu samples with a tunable atomic composition starting from a trimetallic metal mixture. To our knowledge, this has not been described in the literature before.

**Figure 5:**
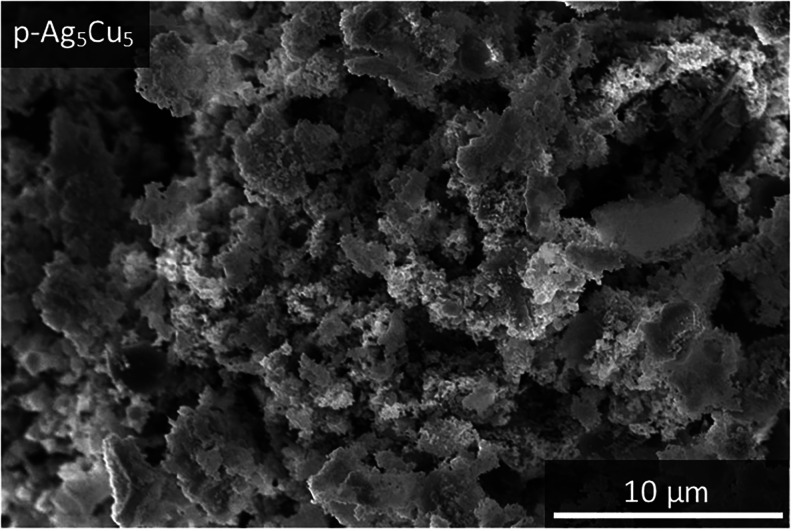
Scanning electron microscopy image of p-Ag_5_Cu_5_.

To quantify the morphology, N_2_-physisorption was measured to determine the pore volume and surface area. The results are listed in Table 2. Surface areas up to 25 m^2^/g were found as well as pore volumes up to 0.11 cm^3^/g. These values are similar to values found in the literature for selectively leached porous metals.^44^ The pore size distribution curves are given in Figure S6, while an overview of the microporosities obtained from the t-plot analysis is given in Table S3. The data shows that all samples are macroporous (>50 nm). On top of that, the samples with a high surface area also show mesopores (2–50 nm) and even a bit of micropores (<2 nm). These results show that it is possible to make high-surface-area bimetallic samples with a tunable composition.

Apart from the porosity, all five samples in Figures 5 and S6 contained nonporous parts of a few microns in size too. Some minor differences were found among the five p-Ag_*x*_Cu_10‑*x*_ samples. The more Cu the samples contained, the less the porous structure looked like a ligament that has been described in the literature before.^3,10^ Instead, structures with facets (such as cubes) become more visible. These cubes are most visible in the p-Cu sample. To be able to relate these shapes to a specific element and to obtain better insight into the distribution of Ag and Cu through the sample, SEM images were combined with energy-dispersive X-ray spectroscopy (EDX) maps. In Figure 6, the EDX map for the p-Ag_5_Cu_5_ is shown. Here, it is clear that down to the scale of a few micrometers, both Ag and Cu are mixed. Further, the SEM–EDX images show that there is still some Al present in the samples. For the other bimetallic samples, similar results were found (see Figures S7 and S8).

**Figure 6:**
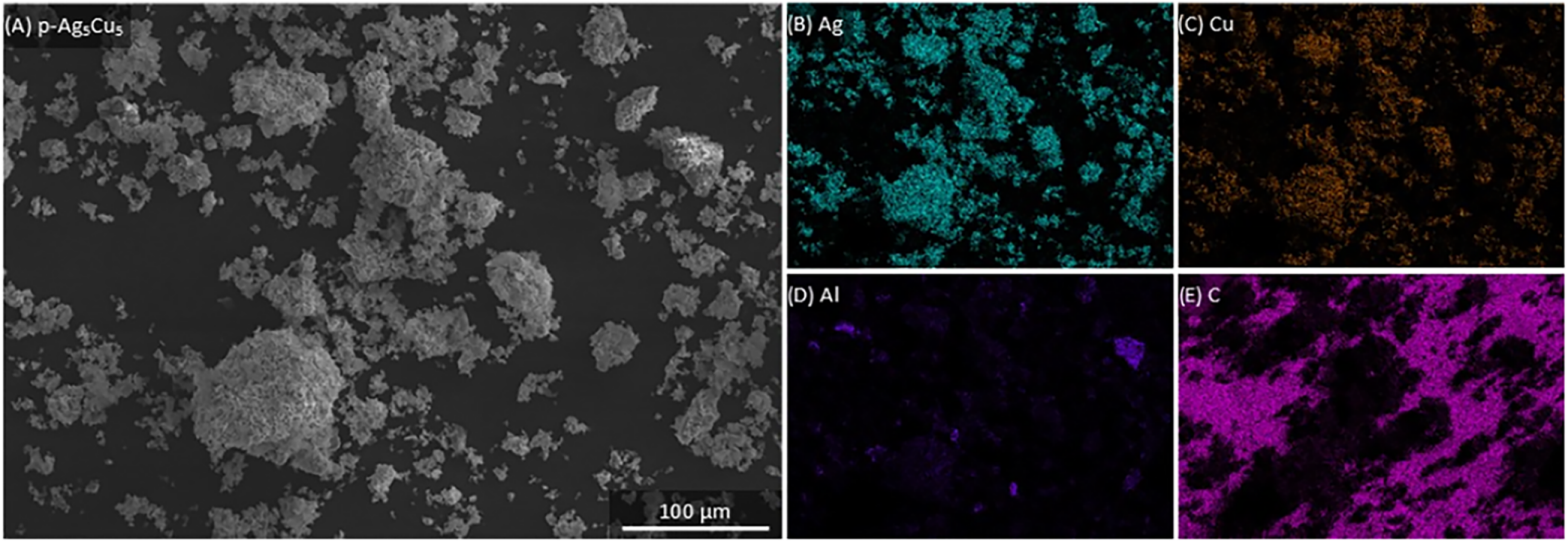
(A) Scanning electron microscopy image of the p-Ag_5_Cu_5_ catalyst and the related energy-dispersive X-ray spectroscopy maps for (B) Ag, (C) Cu, (D) Al, and (E) C.

The SEM–EDX maps show that Ag and Cu are well mixed down to the micrometer scale. To see if they are also mixed on the nanometer scale or even on the atomic scale, scanning transmission electron microscopy (STEM) images in combination with EDX maps were acquired. Figure 7 shows the STEM image and EDX maps for the p-Ag_5_Cu_5_ sample. These images show that down to a scale of tens of nanometers, the Ag and Cu are mixed intimately, creating Ag/Cu interfaces. Similar results were found for the p-Ag_7_Cu_3_ and the p-Ag_3_Cu_7_ samples (see Figures S9 and S10). Hence, this synthesis route is highly suited to make high-surface-area AgCu in various ratios with Ag and Cu in nanoscale proximity.

**Figure 7:**
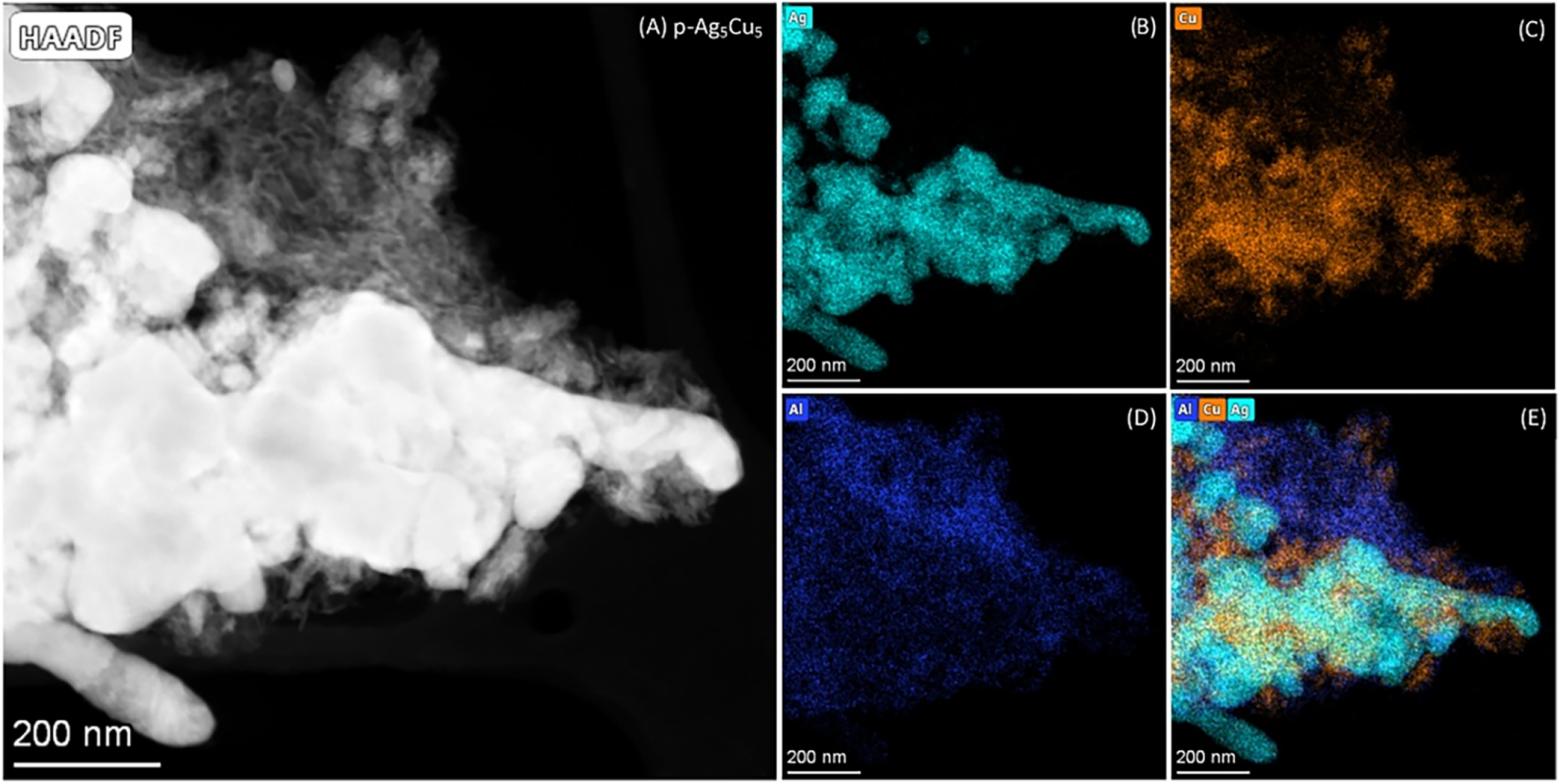
(A) Scanning transmission electron microscopy image of the p-Ag_5_Cu_5_ catalyst and the related energy-dispersive X-ray spectroscopy maps for (B) Ag, (C) Cu, (D) Al, and (E) Ag, Cu, and Al together.

### 3.4 Application: The Electrochemical Reduction of CO_2_

Several applications are known for high-surface-area bimetallics. An interesting example is in electrocatalysis, where the use of bimetallic nanostructures is known to enhance the formation of specific products.^12,45^ Therefore, the dealloyed Ag_*x*_Cu_10‑x_ samples were applied as catalysts in the electrochemical reduction of CO_2_ as a model reaction. This reaction was chosen since Ag and Cu have a very different product selectivity. Ag leads to the production of CO, while Cu can make various products including C_2+_ products.^46^ Combining Ag and Cu might lead to a higher C_2+_ production compared to the pure metals, as CO can be formed on Ag first and then be further converted on Cu.^47–50^

Therefore, this reaction could be used to look at the effect of varying the Ag:Cu ratio. Using the dealloyed p-Ag_*x*_Cu_10‑*x*_ samples, electrodes with a loading of 2.6 mg of Ag_*x*_Cu_10‑*x*_/cm^2^ on carbon paper were made. The double-layer capacitance (Figure S11) showed that the surface area of the whole electrode followed the same trend as the surface area determined via physisorption for the five p-Ag_*x*_Cu_10‑*x*_ samples. The chemical performance of these electrodes was tested by subsequently applying four different potentials (−0.4, −0.6, −0.8, and −1.0 V vs RHE, with 80% *iR* correction during the measurements). The current responses normalized to the geometric surface areas are listed in Figure 8. The current responses strongly differ, despite similar catalyst loadings: the more Cu is present in the sample, the higher the current density. As we will discuss later, this is related to the higher H_2_ formation rate on Cu.

**Figure 8:**
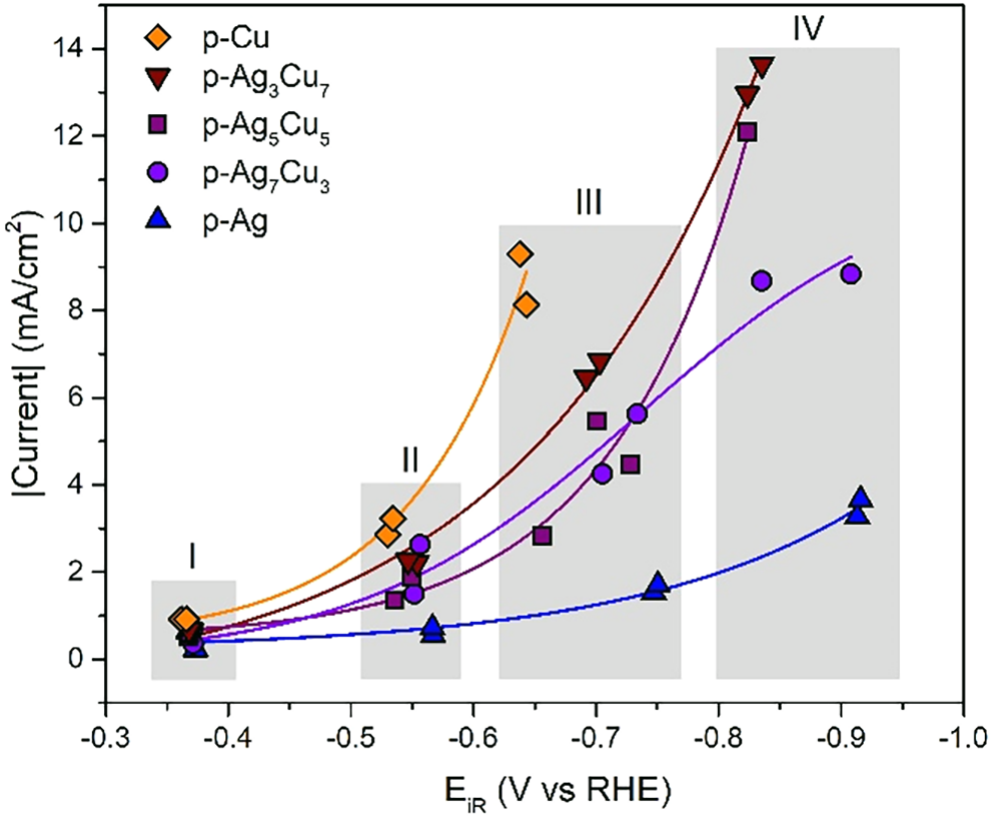
Current densities versus the potential for the p-Ag, p-Ag_7_Cu_3_, p-Ag_5_Cu_5_, p-Ag_3_Cu_7_, and p-Cu catalysts. The gray areas indicated with I, II, III, and IV are the potential groups used in Figure 9. The solid lines serve as a guide for the eye.

It is interesting to look at the selectivity of the catalysts. To the best of our knowledge, there is no literature on the dependence of the selectivity on the Cu content of leached AgCu catalysts. In Figure 9, the partial current densities toward CO, H_2_, C_2_H_4_, and ethanol are given. In Figure 8, we have grouped the data points into 4 groups based on a comparable potential: −0.37, −0.55, −0.70, and −0.87 V vs RHE. In Figure 9, these groups are used to compare the partial current densities for different potentials. Figure 9A shows that for the two least negative potentials (−0.37 and −0.55 V vs RHE), the CO production is almost equal, but for the most negative potentials (−0.70 and −0.87 V vs RHE), the CO production decreases when the Cu content increases. This can be explained by a combination of two effects: first, Cu is less selective to CO than Ag.^51^ At the same time, tandem catalysis could also lead to lower detected CO production.

**Figure 9:**
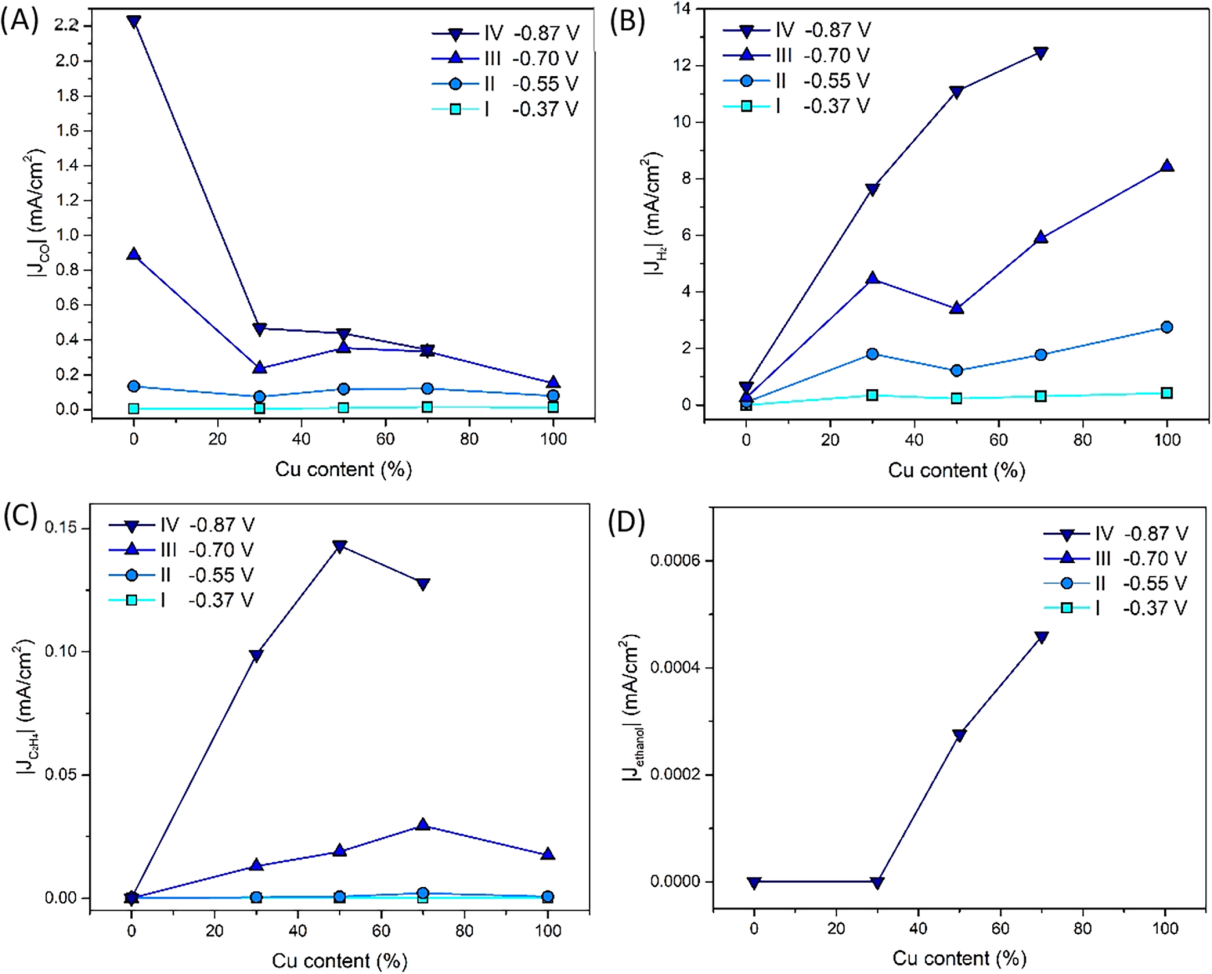
Partial current densities of (A) CO, (B) H_2_, (C) C_2_H_4_, and (D) ethanol versus the Cu content in the porous Ag_*x*_Cu_10‑*x*_ catalyst at four different potentials.

For H_2_ production in Figure 9B, the opposite holds: the more Cu present in the catalysts, the higher the amount of H_2_ formed. This is in line with the higher selectivity of Cu toward H_2_ than Ag.^51^ It is good to note that the current densities to H_2_ are much higher than the partial current densities to any other product (at least 10× higher). So, the differences in total current in Figure 8 are basically the result of differences in H_2_ production. The results in Figure 9B also imply that these catalysts are better catalysts for the hydrogen evolution reaction than for the reduction of CO_2_.

To understand whether there are synergistic effects between Ag and Cu, we can look at the formation of ethylene and ethanol. Figure 9C shows that there is an optimum in the Cu content for the C_2_H_4_ production between 50 and 70 atom % Cu. For nanoparticles, it has been described in the literature before that the interface between Ag and Cu is crucial for the increased formation of C_2_H_4_ and that a higher Cu content than the Ag content is needed to create the most C_2_H_4_.^14^ As we discussed before, the p-Ag_*x*_Cu_10‑*x*_ catalysts have many Ag/Cu interfaces on the nanoscale. Hence, the dependence of C_2_H_4_ production on the presence of Ag/Cu interfaces not only holds for nanoparticles^14^ but can be extended to other bimetallic nanostructures too.

Next, it is interesting to examine the formation of ethanol. Figure 9D shows that only a tiny amount of ethanol was made at the most negative potential. A higher Cu content seems to be better for ethanol production, which is surprising: it is known that ethanol is formed on Cu edges, corners, and subnanometer clusters.^52,53^ Based on the STEM–EDX images in Figures 7, S8, and S9, we conclude that the higher the Cu content, the larger the Cu particles. Hence, there are more terraces that produce C_2_H_4_.^53^ Here, it is worth pointing out that the production of ethanol was very low. So, small fluctuations in the production or the detection with NMR could lead to large differences in the partial current densities.

Finally, we want to point out that after catalysis, SEM­(-EDX), XRD, and ICP data for the p-Ag_*x*_Cu_X‑10_ samples (Figures S12 and S16 and Table S3) looked similar, and only minor amounts of Cu were lost. This is interesting, as most monometallic Cu catalysts for the electrochemical reduction of CO_2_ are known to suffer from restructuring.^54,55^ Hence, these tunable AgCu nanostructures are useful not only to systematically study the effect of Ag:Cu ratio on the activity and selectivity of a chemical reaction but also to offer improved stability.

## 4 Conclusions

In this work, we developed a novel synthesis route for porous bimetallic AgCu catalysts by leaching Al from trimetallic AgCu metal mixtures. We introduced a quenching step to suppress the formation of Ag_2_Al and CuAl_2_ phases. For the first time, we have shown that this synthesis route provides good control over the composition of porous Ag_*x*_Cu_10‑*x*_ over the full range from *x* = 0 to *x* = 10. These porous AgCu catalysts consist of Ag- and Cu-based phases, which are not alloyed but in close proximity to each other (tens of nanometers). The metal mixtures were used in the electrochemical reduction of CO_2_ as a cathode material. Here, we have shown that the selectivity depends on the Cu content. Especially, an optimum in C_2_H_4_ production was found for a Cu content between 50 and 70 atom % in the nanostructures. On top of that, the catalysts did not change significantly, showing good stability. This shows the applicability of the novel synthesis route covering the full Ag:Cu ratio.

## Supplementary Material



## Notes

1. Rietveld refinement; 2. X-ray diffractograms; 3. inductively coupled plasma; 4. X-ray photoelectron spectroscopy; 5. scanning electron microscopy; 6. pore size distribution; 7. scanning electron microscopy with energy-dispersive X-ray spectroscopy; 8. scanning transmission electron microscopy with energy-dispersive X-ray spectroscopy; 9. double-layer capacitance; 10. X-ray diffractograms after catalysis; 11. scanning electron microscopy after catalysis; 12. inductively coupled plasma after catalysis; and 13. scanning electron microscopy with energy-dispersive X-ray spectroscopy after catalysis (PDF)
