# Supplementary material for: Design of High-Surface-Area Bimetallic Ag–Cu Nanostructures with a Tunable Ratio Obtained via Selective Leaching of AlAgCu Alloys
Source: J Phys Chem C Nanomater Interfaces. 2025 Jul 24;129(31):13961–70. doi: 10.1021/acs.jpcc.5c02677 (PMC12337803; doi:10.1021/acs.jpcc.5c02677)
Supplement: Supplementary file 1 [file jp5c02677_si_001.pdf]

SI: Design of High-Surface-Area Bimetallic Ag-Cu Nanostructures with a Tunable Ratio Obtained *via* Selective Leaching of AlAgCu Alloys

Maaike E.T. Vink - van Ittersum[1], Masoud Lazemi[1], Remco Dalebout[1], Johannes D. Meeldijk[1], Matt L. J. Peerlings[1], Juliette C. Verschoor[1], Bianca Ligt[2], Emiel Hensen[2], Ad van der Eerden[1], Peter Ngene[1] and Petra E. de Jongh[1*]

[1]*Materials Chemistry & Catalysis, Debye Institute for Nanomaterials Science, Utrecht University, Universiteitsweg 99, 3584CG Utrecht, The Netherlands*

[2]*Inorganic Materials & Catalysis, Chemical Engineering and Chemistry, Eindhoven University of Technology*

**corresponding author: p.e.dejongh@uu.nl*

Contents

1. Rietveld refinement
2. X-ray diffractograms
3. Inductively couple plasma
4. X-ray photoelectron spectroscopy
5. Scanning electron microscopy
6. Pore size distribution
7. Scanning electron microscopy with energy-dispersive X-ray spectroscopy
8. Scanning transmission electron microscopy with energy-dispersive X-ray spectroscopy
9. Double-layer capacitance
10. X-ray diffractograms after catalysis
11. Scanning electron microscopy after catalysis
12. Inductively couple plasma after catalysis
13. Scanning electron microscopy with energy-dispersive X-ray spectroscopy after catalysis

1. Rietveld refinement

Table S 1. *Percentages of crystalline phases based on Rietveld refinement of the X-ray diffractograms using the Bruker DIFFRAC.SUITE TOPAS software. The crystallographic data of Al (cubic), Ag (cubic), Cu (cubic), Ag_2Al (hexagonal), $CuAl_2$ (tetragonal), Cu_2O (cubic) and CuO (monoclinic) between 40 ° and 120 ° 2θ (Co source) or between 33 ° and 97 ° 2θ (Cu source) was fitted using a Lorentzian function for its peaks. No strain was incorporated in the lattice planes, but preferred orientations were used as expected for the non-powdered samples. The background was fitted with a Chebyshev polynomial with $n \leq 3$. The composition based on the crystalline phases present was obtained after the fitting reached a minimum goodness-of-fit (GOF) value.*

Sample	Al (at%)	Ag (at%)	Cu (at%)	Ag_2Al (at%)	$CuAl_2$ (at%)	Cu_2O (at%)	CuO (at%)
$Al_{90}Ag_5Cu_5$ phys. mix.	84.6	9.2	6.2	-	-	-	-
$Al_{90}Ag_5Cu_5$ alloyed	81.8	-	-	3.4	14.8	-	-
$Al_{90}Ag_5Cu_5$ quenched	94.6	-	-	0.9	4.4	-	-
p-Ag_5Cu_5	-	96.6	-	-	-	3.4	-
p-Ag	-	100.0	-	-	-	-	-
p-Ag_7Cu_3	-	99.2	-	-	-	0.8	-
p-Ag_3Cu_7	-	78.0	0.2	-	-	21.9	-
p-Cu	-	-	0.1	-	-	10.0	89.9

A) $Al_{90}Ag_5Cu_5$ physical mixture

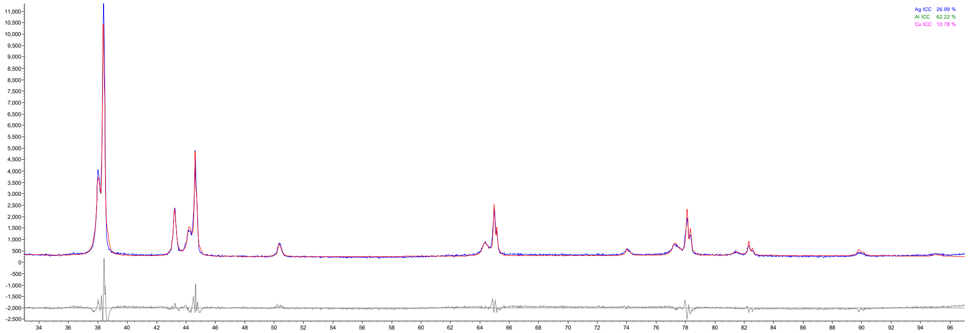

B) $Al_{90}Ag_5Cu_5$ alloyed

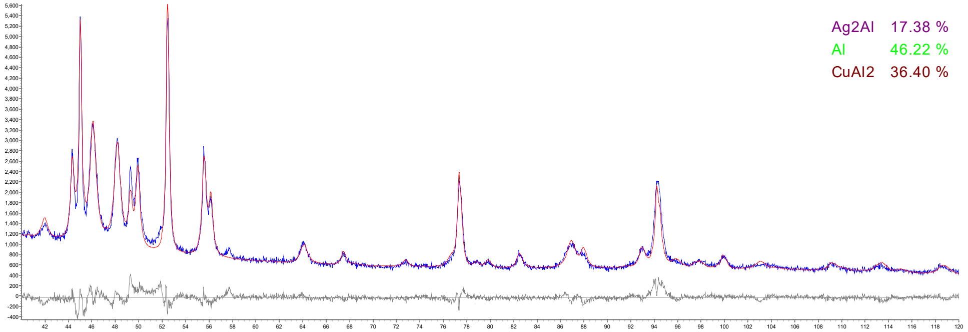

C) $Al_{90}Ag_5Cu_5$ quenched

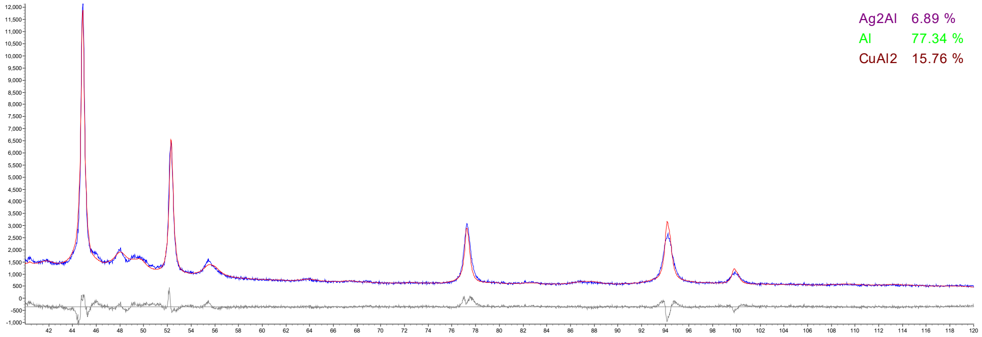

D) p-Ag_5Cu_5

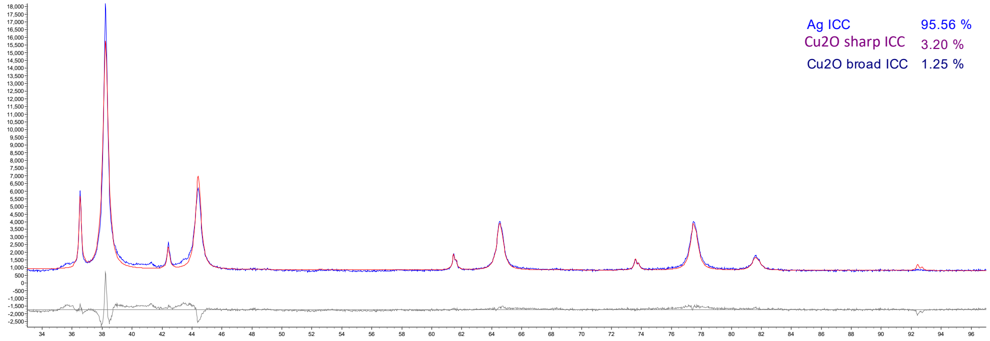

E) p-Ag

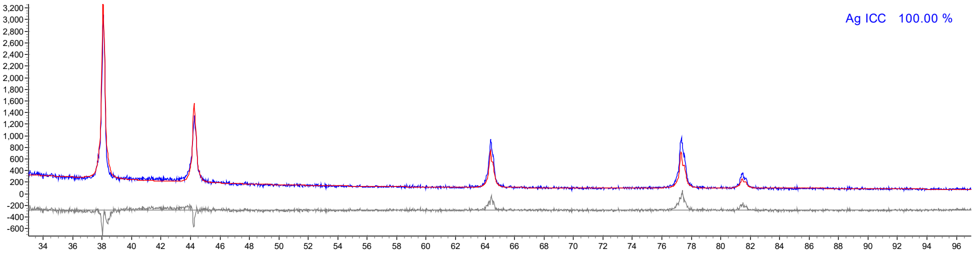

F) p-Ag_7Cu_3

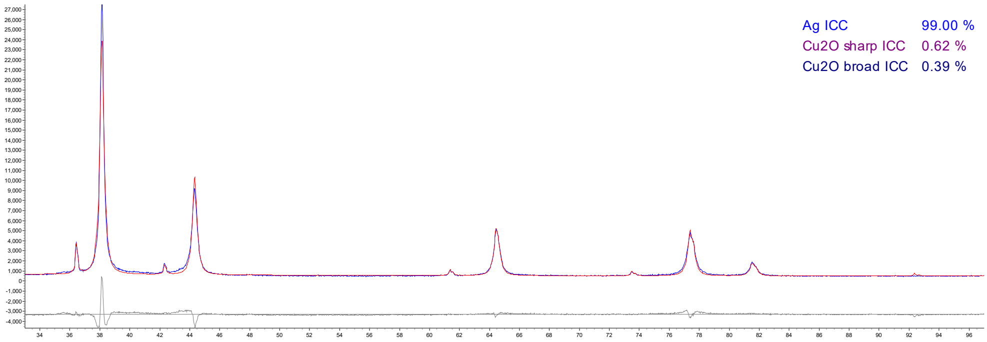

G) p-Ag_3Cu_7

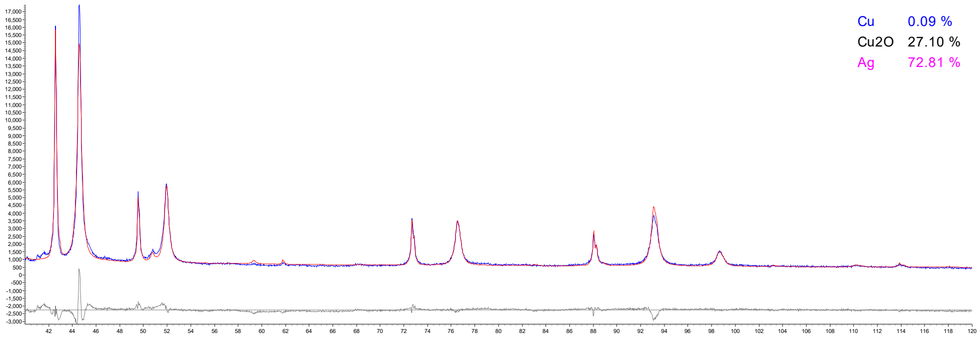

H) p-Cu

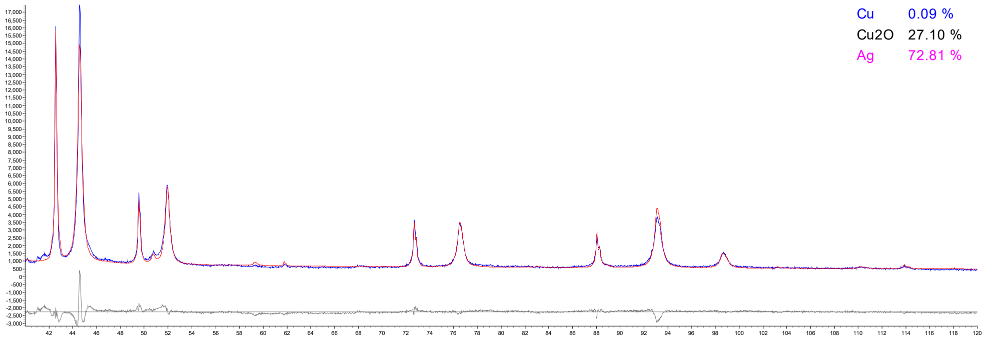

Figure S 1. *Fitting of the X-ray diffractograms using Rietveld refinement in the Bruker DIFFRAC.SUITE TOPAS software for a) the physical mixture $Al_{90}Ag_5Cu_5$; b) alloyed $Al_{90}Ag_5Cu_5$; c) quenched $Al_{90}Ag_5Cu_5$; d) p-Ag_5Cu_5; e) p-Ag; f) p-Ag_7Cu_3; g) p-Ag_3Cu_7; and h) p-Cu.*

2. X-ray diffractograms

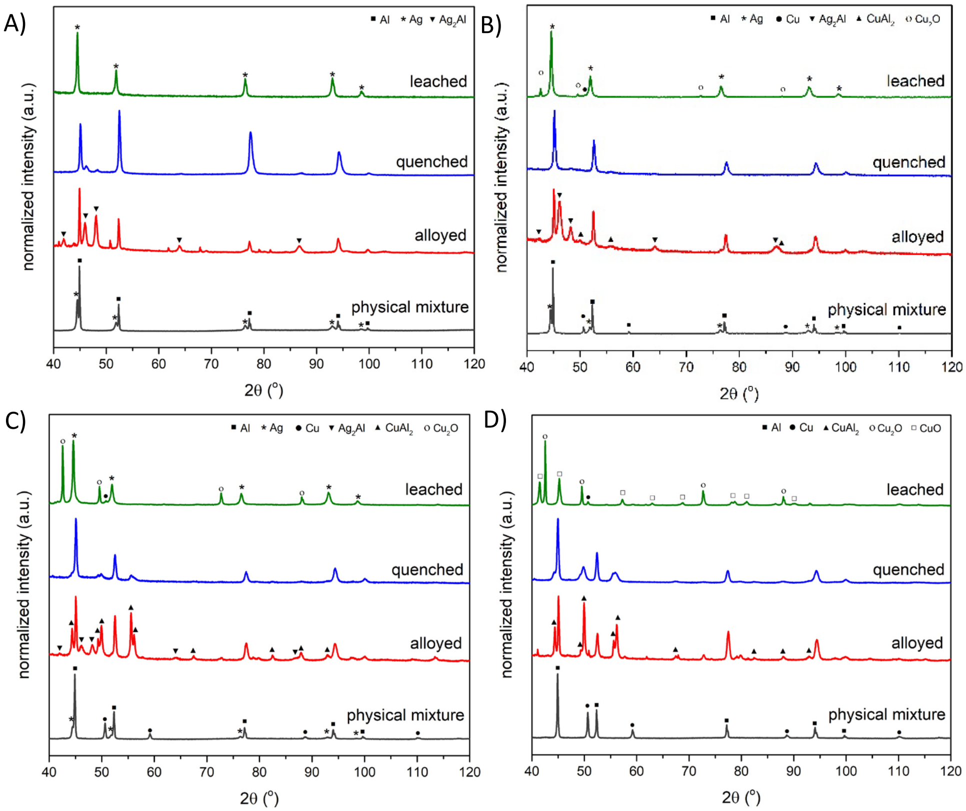

Figure S 2. *X-ray diffraction patterns for the physical mixture, alloyed mixture, quenched mixture and leached mixture of a) $(Al_{90})Ag_{10}$; b) $(Al_{90})Ag_7Cu_3$; c) $(Al_{90})Ag_3Cu_7$ and d)$(Al_{90})Cu_{10}$ measured on a Bruker D2 Phaser with a Co Kα X-ray source (1.79028 Å) and on a Bruker D2 Phaser with a Cu Kα X-ray source (1.54187 Å) with a step size of 0.03 ° and a dwell time of 1 s.*

3. Inductively coupled plasma

Table S2. *ICP of the dealloyed p-Ag_xCu_{10-x} samples.*

Sample	*Ag (wt%)*	*Cu (wt%)*	*Al (wt%)*	*C (wt%)*	*Total (wt%)*	*Weight ratio Ag:Cu*	*Atomic ratio*
p-Ag	96.71	0.02	0.59	0.19	97.51	-	Ag_{10}
p-Ag_7Cu_3	74.04	18.01	1.51	0.35	93.91	4.11	$Ag_{7.08}Cu_{2.92}$
p-Ag_5Cu_5	59.02	31.64	3.70	0.51	94.87	1.87	$Ag_{5.24}Cu_{4.76}$
p-Ag_3Cu_7	38.90	53.79	2.56	0.37	95.62	0.72	$Ag_{2.99}Cu_{7.01}$
p-Cu	0.02	96.12	1.15	0.17	97.46	-	Cu_{10}

4. X-ray photoelectron spectroscopy

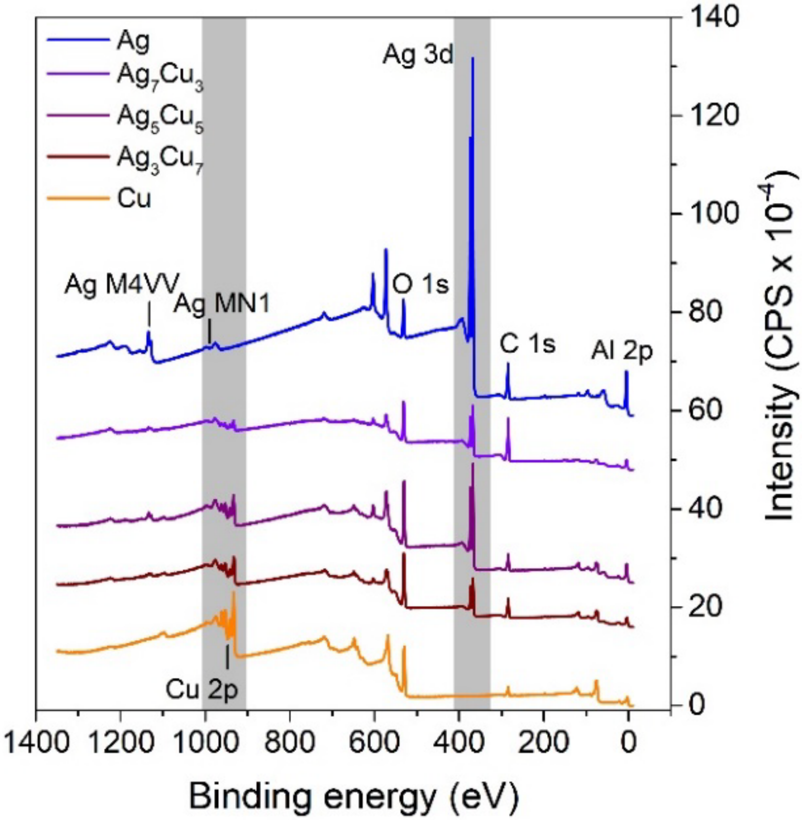

Figure S 3. *X-ray photoelectron spectroscopy of p-Ag, p-Ag_7Cu_3, p-Ag_5Cu_5, p-Ag_3Cu_7 and p-Cu measured on a K-Alpha ultra-high vacuum X-ray photoelectron spectrometer (Thermo Fisher Scientific) using a monochromatic aluminum anode (Kα = 1486.6 eV, 72 W) X-ray source with a spot size of 400 µm. The samples were measured with a pass energy of 50 eV.*

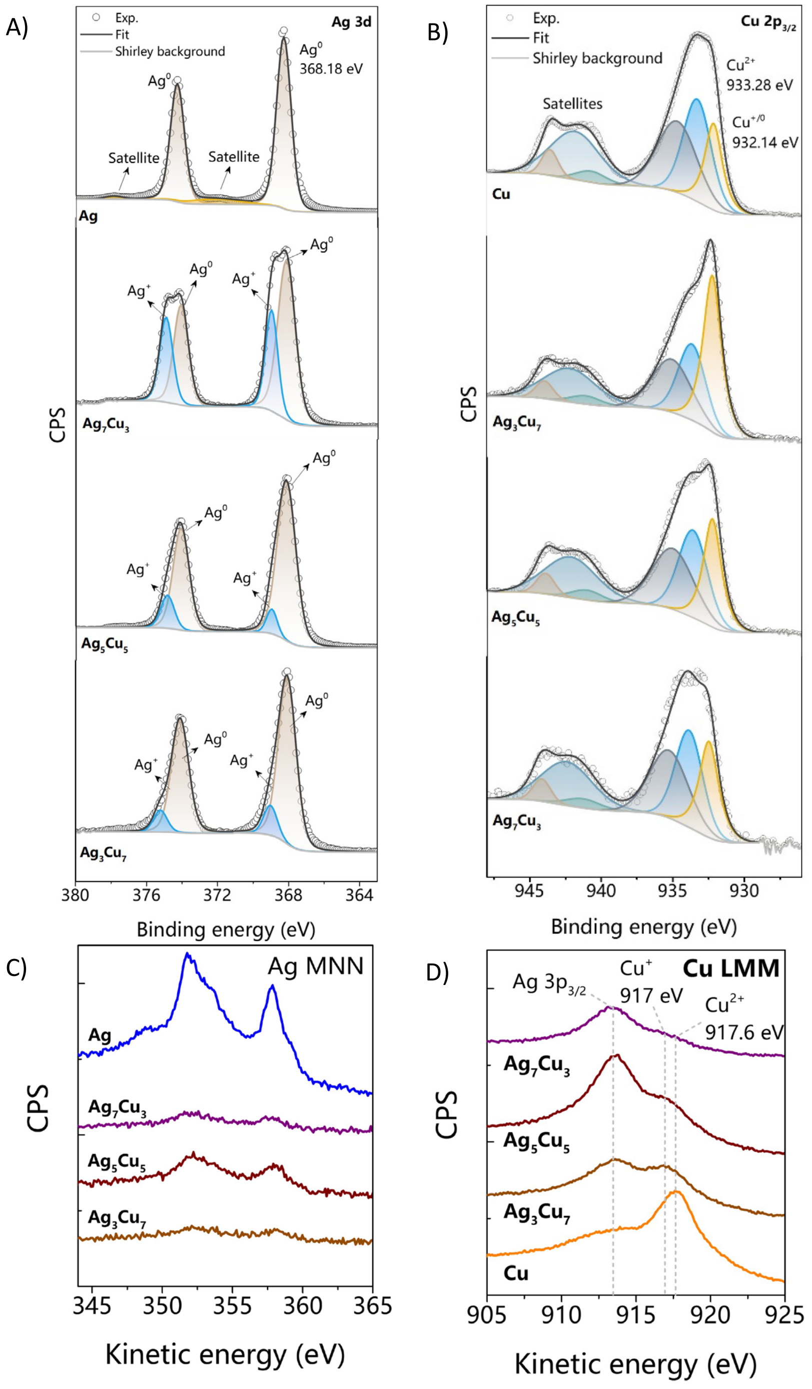

Figure S 4. *Fitting of the a) Ag XPS peaks and b) the Cu XPS peaks for p-Ag, p-Ag_7Cu_3, p-Ag_5Cu_5, p-Ag_3Cu_7 and p-Cu using peak positions described in literature; there is a slight variation in binding energy positions which we ascribe to slight charging of the samples and/or some residual Al in the Ag and Cu phases and c) Ag MNN and d) Cu LMM peaks to determine which oxidation states of Ag and Cu are present.*

5. Scanning electron microscopy

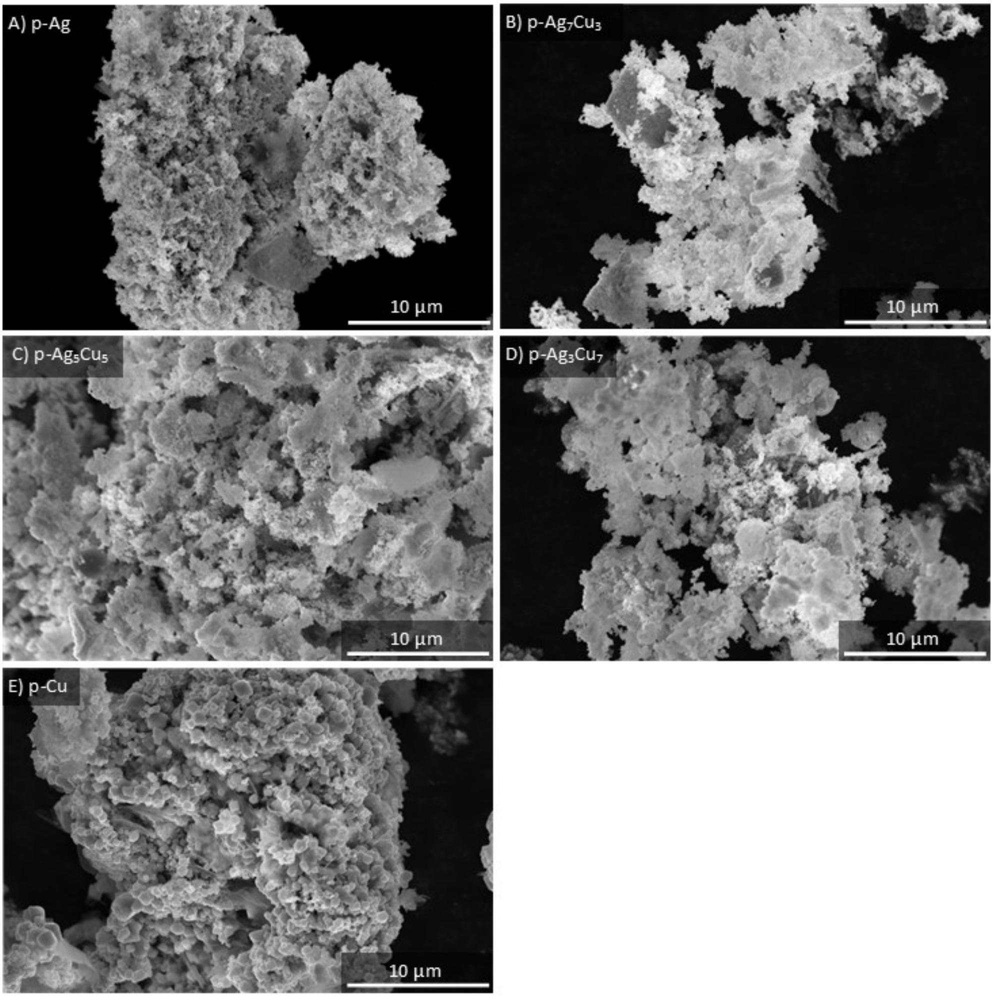

Figure S 5. *Scanning electron microscopy images of a) p-Ag; b) p-Ag_7Cu_3; c) p-Ag_5Cu_5; d) p-Ag_3Cu_7 and e) p-Cu. The images were taken on a Thermo Fisher Scientific Helios G3 UC operated at 15 kV and 50 pA.*

6. Pore size distribution

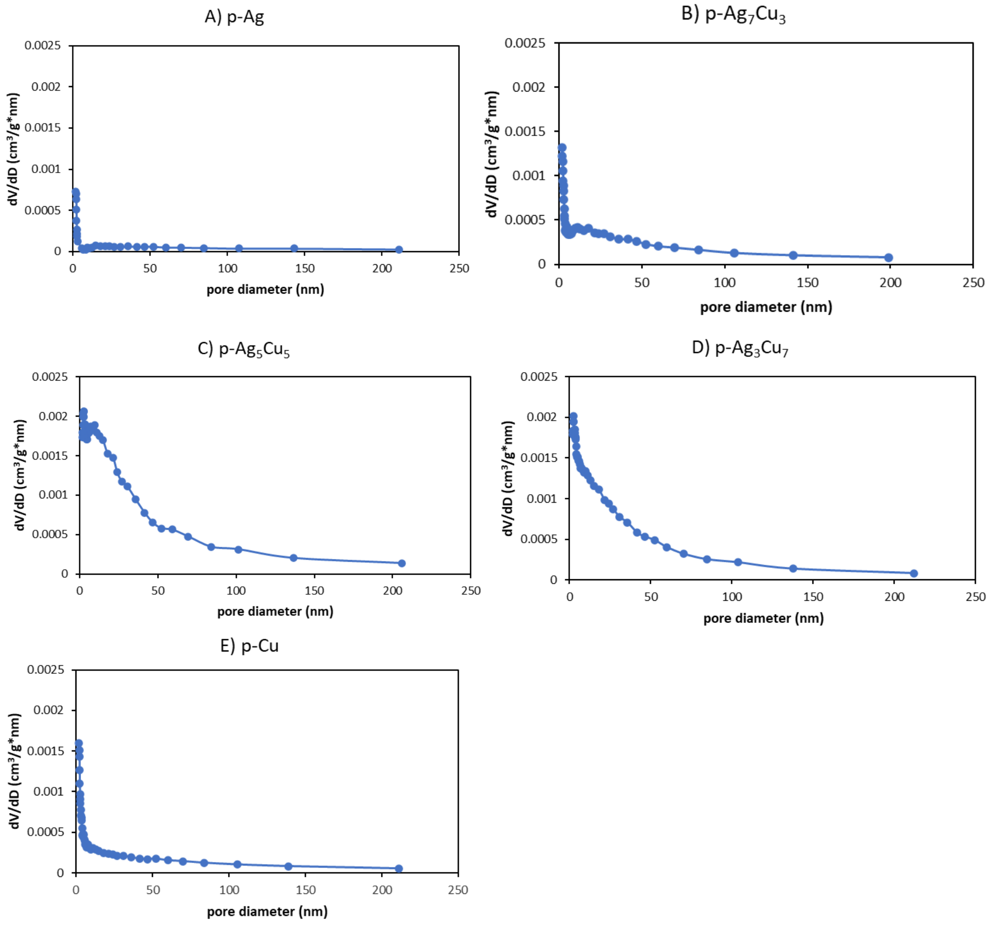

Figure S 6. *Differential volumes over pore diameter based on BJH analysis of the adsorption branch of the physisorption data for a) p-Ag; b) p-Ag_7Cu_3; c) p-Ag_5Cu_5; d) p-Ag_3Cu_7 and e) p-Cu.*

Table S3. *Micropore volume based on the t-plot.*

Sample	*Micropore volume (cm^3/g)*
p-Ag	-
p-Ag_7Cu_3	0.000132
p-Ag_5Cu_5	0.000655
p-Ag_3Cu_7	0.001233
p-Cu	0.000789

7. Scanning electron microscopy energy-dispersive X-ray spectroscopy

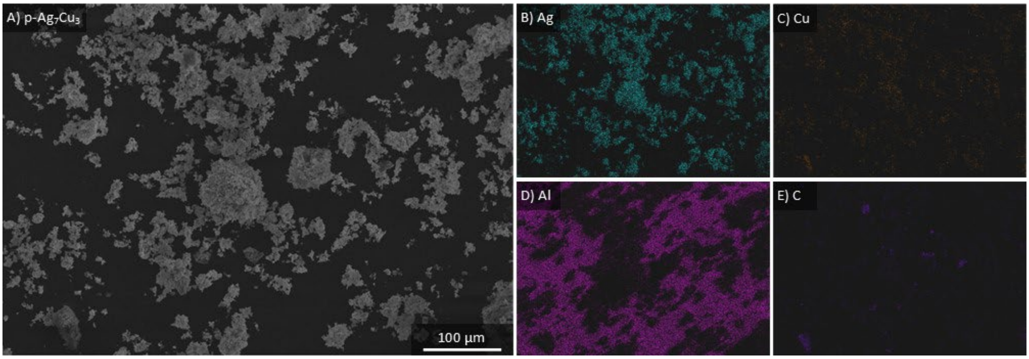

Figure S 8. *a) Scanning electron microscopy image of the p-Ag_7Cu_3 catalyst and the related energy-dispersive X-ray spectroscopy maps for b) Ag; c) Cu; d) Al and e) C. The image was taken on a Thermo Fisher Scientific Helios G3 UC operated at 15 kV and 50 pA equipped with an Oxford Instruments X-Max^N 150 mm^2 detector for EDX measurements.*

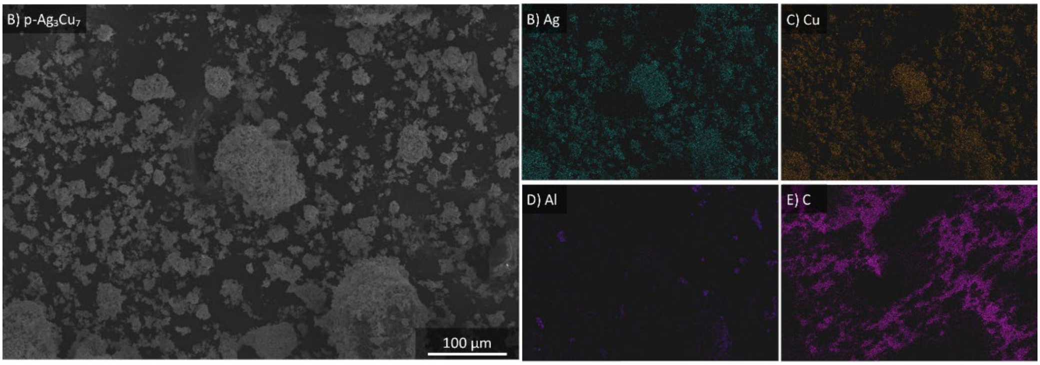

Figure S 7. *a) Scanning electron microscopy image of the p-Ag_3Cu_7 catalyst and the related energy-dispersive X-ray spectroscopy maps for b) Ag; c) Cu; d) Al and e) C. The image was taken on a Thermo Fisher Scientific Helios G3 UC operated at 15 kV and 50 pA equipped with an Oxford Instruments X-Max^N 150 mm^2 detector for EDX measurements.*

8. Scanning transmission electron microscopy energy-dispersive X-ray spectroscopy

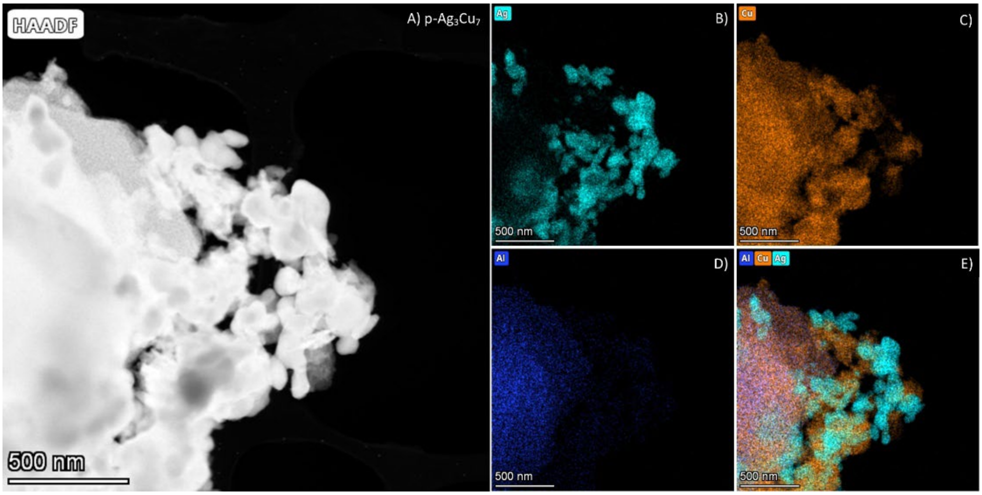

Figure S 10. *Scanning transmission electron microscopy image of the p-Ag_3Cu_7 catalyst and the related energy dispersive X-ray spectroscopy maps for b) Ag; c) Cu; d) Al and e) Ag, Cu and Al together. The image and EDX map were taken using a Talos F200x at 200 kV microscope equipped with a super XG1 EDX detector in a low background TEM holder using the net signal in the Velox software.*

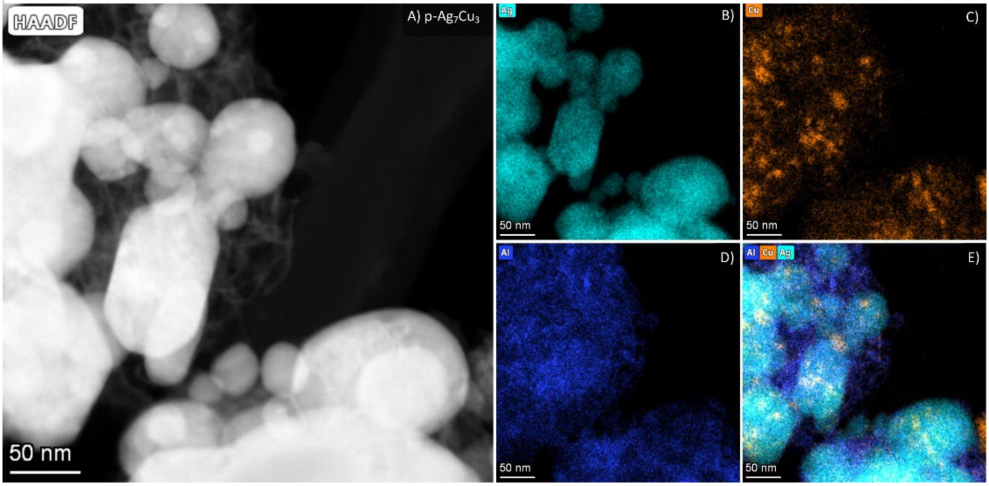

Figure S 9*. Scanning transmission electron microscopy image of the p-Ag_7Cu_3 catalyst and the related energy- dispersive X-ray spectroscopy maps for b) Ag; c) Cu; d) Al and e) Ag, Cu and Al together. The image and EDX map were taken using a Talos F200x at 200 kV microscope equipped with a super XG1 EDX detector in a low background TEM holder using the net signal in the Velox software.*

9. Double-layer capacitance

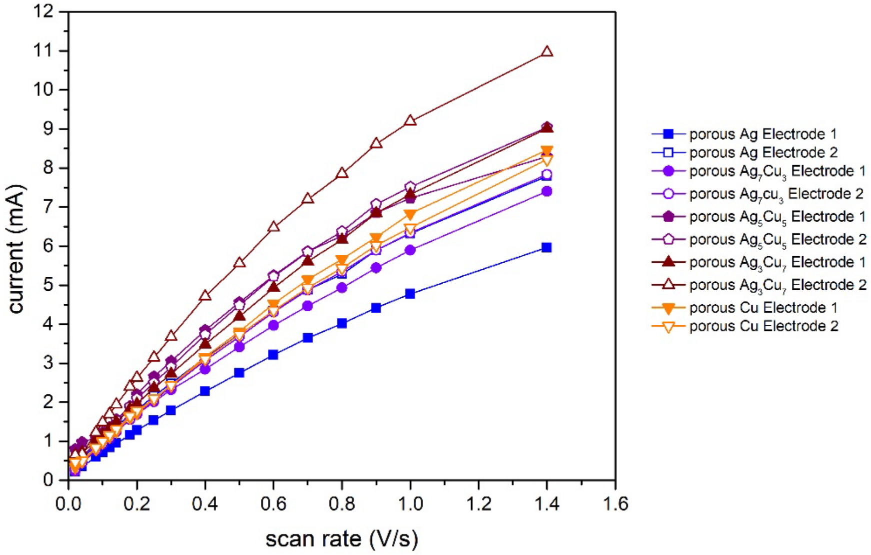

Figure S 11. *Current vs scan rate plots for the determination of the double-layer capacitance of the porous* Ag_xCu_{10-x} *samples.*

10. X-ray diffractograms after catalysis

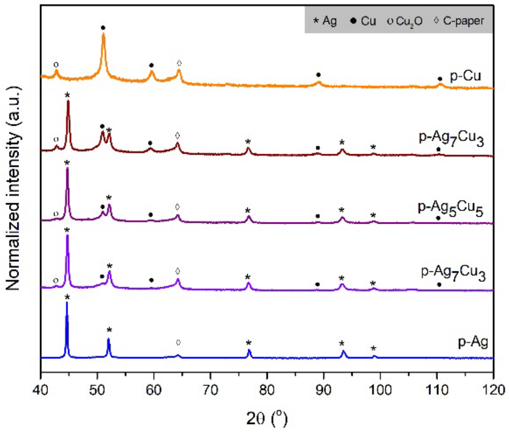

Figure S 12. *X-ray diffractograms of p-Ag, p-*Ag_7Cu_3*, p-*Ag_5Cu_5*, p-*Ag_3Cu_7 *and p-Cu electrodes after catalysis measured on a Bruker D2 Phaser with a Cu Kα X-ray source (1.54187 Å) with a step size of 0.03 ° and a dwell time of 1 s.*

11. Scanning electron microscopy after catalysis

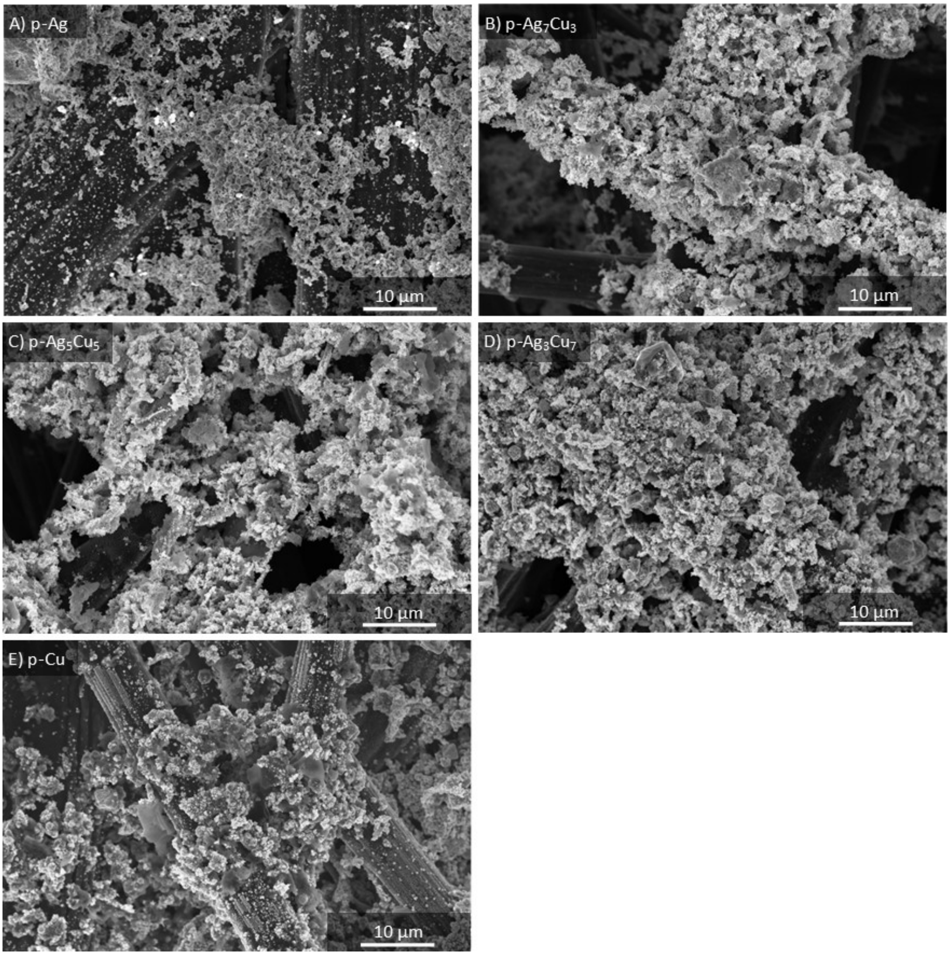

Figure S 13. *Scanning electron microscopy images after catalysis of a) p-Ag; b) p-Ag_7Cu_3; c) p-Ag_5Cu_5; d) p-Ag_3Cu_7 and e) p-Cu. The images were taken on a Thermo Fisher Scientific Helios G3 UC operated at 10 kV and 50 pA.*

12. Inductively couple plasma after catalysis

Table S 3. *ICP of the p-Ag_xCu_{10-x} electrodes after catalysis*

Sample	*Ag (wt%)*	*Cu (wt%)*	*Al (wt%)*	*C (wt%)*	*Total (wt%)*	*Weight ratio Ag:Cu*	*Atomic ratio*
p-Ag	8.35	-	0.18	90.69	99.22	-	Ag_{10}
p-Ag_7Cu_3	6.80	1.65	0.10	89.86	98.41	4.12	$Ag_{7.08}Cu_{2.92}$
p-Ag_5Cu_5	6.33	3.69	0.35	89.05	99.42	1.72	$Ag_{5.03}Cu_{4.97}$
p-Ag_3Cu_7	5.04	7.40	0.23	86.34	99.01	0.68	$Ag_{2.86}Cu_{7.14}$
p-Cu	-	9.43	0.17	88.68	98.28	-	Cu_{10}

13. Scanning electron microscopy energy-dispersive X-ray spectroscopy after catalysis

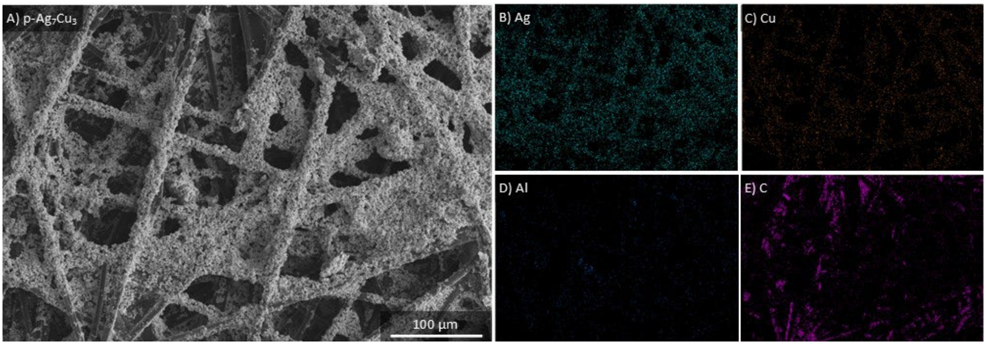

Figure S 14. *a) Scanning electron microscopy image of the p-Ag_7Cu_3 catalyst after catalysis and the related energy-dispersive X-ray spectroscopy maps for b) Ag; c) Cu; d) Al and e) C. The image was taken on a Thermo Fisher Scientific Helios G3 UC operated at 10 kV and 50 pA equipped with an Oxford Instruments X-Max^N 150 mm^2 detector for EDX measurements.*

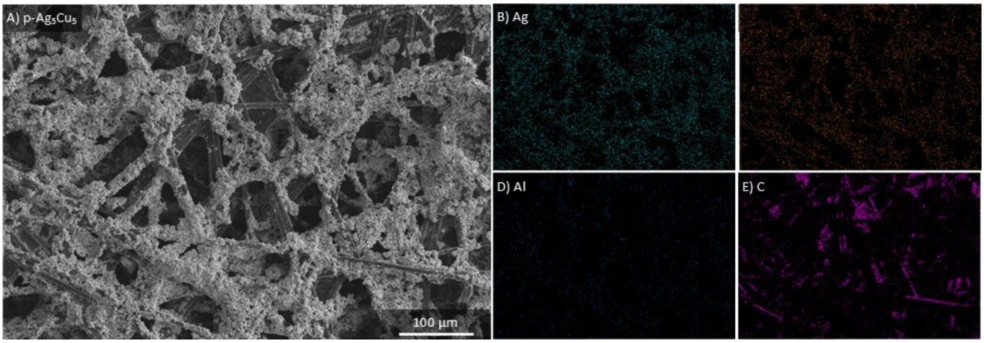

Figure S 15. *a) Scanning electron microscopy image of the p-Ag_5Cu_5 catalyst after catalysis and the related energy-dispersive X-ray spectroscopy maps for b) Ag; c) Cu; d) Al and e) C. The image was taken on a Thermo Fisher Scientific Helios G3 UC operated at 10 kV and 50 pA equipped with an Oxford Instruments X-Max^N 150 mm^2 detector for EDX measurements.*

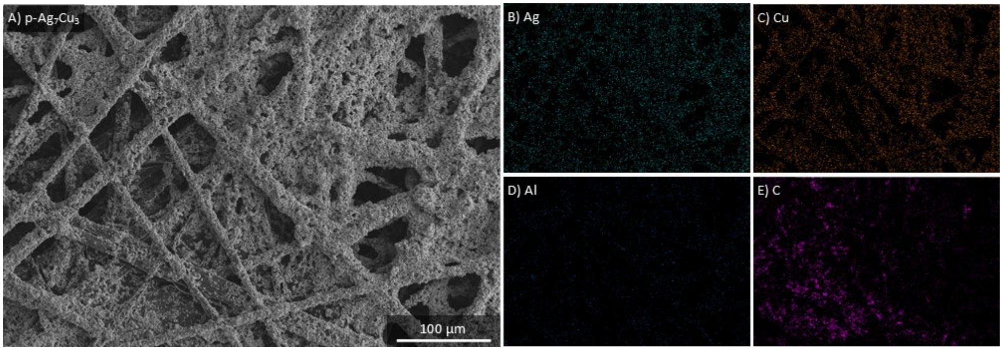

Figure S 16. *a) Scanning electron microscopy image of the p-Ag_3Cu_7 catalyst after catalysis and the related energy-dispersive X-ray spectroscopy maps for b) Ag; c) Cu; d) Al and e) C. The image was taken on a Thermo Fisher Scientific Helios G3 UC operated at 10 kV and 50 pA equipped with an Oxford Instruments X-MaxN 150 mm^2 detector for EDX measurements.*
